# Autoencoder-Based FM Spectrum Anomaly Detection: A Pipeline-Level Study of Preprocessing, Model Capacity, and Reconstruction Scoring

**DOI:** 10.3390/s26185979

**Published:** 2026-09-21

**Authors:** Szilárd László Takács, Konrád Kajdy

**Affiliations:** Department of Electrical Engineering and Info-Communications, Faculty of Informatics and Electrical Engineering, Széchenyi István University, Egyetem tér 1, 9026 Győr, Hungary; kajdykonrad@gmail.com

**Keywords:** radio spectrum monitoring, anomaly detection, convolutional autoencoder, reconstruction error, one-class anomaly detection, VHF Band II, radio broadcasting, TopK scoring

## Abstract

Automated anomaly detection is increasingly important in radio spectrum monitoring, where large volumes of continuously generated measurements must be analyzed while anomalous events remain relatively rare. This study investigates reconstruction-based anomaly detection for real-world very-high-frequency (VHF) Band II radio-broadcasting spectrum measurements using autoencoders whose gradient-based parameter optimization was performed exclusively on normal observations. Measurements covering 87.5–108 MHz in VHF Band II were represented as time–frequency images and processed through a unified pipeline comprising preprocessing, representation learning, reconstruction, residual-based scoring, and anomaly decision. The effects of input normalization, image resolution, network depth, latent-space dimensionality, encoder–decoder symmetry, spectrum representation, and reconstruction scoring were systematically evaluated across symmetric, asymmetric, stacked, augmented stacked, and pixel-sorted autoencoder configurations, while exploratory CVAE (convolutional variational autoencoder) experiments were additionally used to examine probabilistic latent regularization. Increasing architectural complexity did not necessarily improve detection performance. The best configuration identified during model development achieved an $F_{1}$-score of 91.4% on the labeled development subset at its $F_{1}$-optimized operating point. To obtain an independent assessment of generalization, the finalized pipelines were subsequently evaluated on a separate held-out test subset that was excluded from model, checkpoint, scoring-hyperparameter, and threshold selection. The held-out evaluation showed lower absolute performance and a change in configuration ranking, demonstrating that development-set differences should not be interpreted as definitive evidence of architectural superiority. A controlled reconstruction-scoring ablation further showed that anomaly separability depends materially on the formulation of the observation-level reconstruction score.

## 1. Introduction

The radio-frequency spectrum (RF) is a limited natural resource whose efficient, regulated, and interference-free use is essential for the reliable operation of modern wireless communication systems. The continuous evolution of wireless services and communication technologies, together with the growing demand for spectrum resources, has further increased the importance of spectrum monitoring. Modern monitoring systems must process large volumes of continuously generated measurements and identify events that deviate from expected patterns of usage of the spectrum. In Hungary, the nationwide spectrum-monitoring network enables systematic and long-term observation of the RF environment and supports the construction of large-scale measurement datasets suitable for the development and evaluation of automated spectrum-analysis methods, including machine-learning-based anomaly detection.

A particularly informative output of spectrum-monitoring systems is the waterfall representation, in which frequency, time, and received signal intensity are jointly represented in a two-dimensional form. Consequently, spectrum-monitoring tasks can also be approached using image-based analysis techniques. However, real-world waterfall measurements exhibit several characteristics that complicate automated anomaly detection. Measurements acquired at different monitoring stations can vary considerably in absolute signal level, number of active transmitters, intensity distribution, and noise characteristics. Moreover, measurement noise may influence a substantial fraction of an observation, whereas an operationally relevant anomaly may affect only a small spatial or temporal region. The present study considers waterfall measurements covering the 87.5–108 MHz portion of VHF Band II used for radio broadcasting and focuses on events such as transmission interruptions, loss of modulation, and abnormal frequency-related changes.

A fundamental difficulty in anomaly detection is the strong imbalance between normal and abnormal observations. Genuine abnormal events occur substantially less frequently than normal operating conditions, making it difficult to construct sufficiently large and representative anomaly datasets for supervised training. In spectrum-monitoring applications, this difficulty is further amplified because reliable annotation generally requires domain expertise. Milosheski et al. [1], in their investigation of self-supervised representation learning for wireless spectrum activity, highlighted the cost and difficulty associated with manually labeling real-world spectrum observations. More generally, the scarcity, diversity, and incomplete coverage of anomalous examples are recognized as central challenges in deep anomaly detection [2].

These characteristics motivate approaches that do not require representative examples of every possible type of anomaly during training. Reconstruction-based autoencoders provide a natural framework for this setting. An encoder maps an input observation to a lower-dimensional latent representation, while a decoder attempts to reconstruct the original input. When training is restricted to normal observations, the model learns representations and reconstruction patterns associated primarily with normal operating conditions. Observations that deviate from this learned distribution can then produce increased or structurally different reconstruction residuals, which may be used as indicators of anomalous behavior. Reconstruction-based models therefore constitute an important family of deep anomaly-detection methods [2].

Previous RF anomaly-detection studies have demonstrated the applicability of autoencoder-based methods across power spectral density, in-phase/quadrature (IQ), and time–frequency representations [3,4,5,6]. However, these studies differ substantially in their signal representations, frequency environments, architectures, and anomaly definitions, making it difficult to isolate how preprocessing, reconstruction capacity, encoder–decoder design, and anomaly-score formulation interact in noisy operational VHF Band II waterfall measurements.

Our previous work established the experimental basis for addressing this problem in two consecutive stages. First, a measurement methodology, annotation procedure, and measurement-based benchmark dataset were introduced for the VHF Band II broadcasting spectrum [7]. The corresponding measurements are also publicly available as a re-usable dataset [8]. Covering the FM band, this benchmark provides a reproducible basis for the development and comparison of automated spectrum-analysis methods. In a subsequent study, the architectures of vanilla autoencoder (AE), convolutional autoencoder (CAE), and variational autoencoder (VAE) were evaluated in waterfall representations, together with supervised classifiers operating on encoder-derived latent features [9]. The standalone reconstruction-based models achieved $F_{1}$-scores between 79.4% and 81.3%, while the combination of a CAE encoder and an ExtraTrees classifier achieved an $F_{1}$-score of 91.5%. Although these results demonstrated the potential of learned representations for VHF Band II broadcasting-spectrum anomaly detection, they did not systematically investigate the individual design factors that govern the performance of a reconstruction-based detector whose gradient-based parameter updates are computed exclusively from normal observations.

This distinction leads to the central research question of the present study: which components of a reconstruction-based anomaly-detection pipeline most strongly determine detection performance on noisy, real-world FM waterfall measurements? Performance is potentially influenced not only by the selected autoencoder family, but also by input normalization and resolution, network depth and capacity, latent dimensionality, encoder–decoder symmetry, input representation, and, critically, the method used to transform a high-dimensional reconstruction residual into a scalar anomaly score. The importance of the reconstruction criterion has also been demonstrated in image-based anomaly-detection applications. Bergmann et al. [10], for example, investigated structural similarity for unsupervised defect segmentation using autoencoders and demonstrated that structurally informed reconstruction criteria can behave differently from conventional pixel-wise error measures.

The anomaly-score formulation is particularly important for the investigated VHF Band II waterfall measurements because relevant anomalies may occupy only a small localized region, while measurement noise can be distributed across a substantial portion of an image. Consequently, globally aggregated reconstruction measures may dilute diagnostically relevant localized residuals, motivating the evaluation of scoring strategies that preserve prominent reconstruction discrepancies.

Therefore, the present study treats VHF Band II broadcasting-spectrum anomaly detection based on autoencoders as an integrated processing pipeline rather than as an isolated neural-network architecture. Our experiments systematically examined the effects of input preprocessing and normalization, image resolution, network depth, latent-space dimensionality, encoder–decoder symmetry, alternative convolutional structures, variational representations, edge-enhanced inputs, and spectrum-specific pixel transformations. In parallel, the anomaly-score formulation was examined as an additional design dimension across the evaluated model–scoring configurations by comparing global MSE and structural similarity (SSIM), maximum reconstruction residuals, percentile and dispersion-based statistics, and aggregation strategies that retained the largest localized reconstruction errors. All reconstruction-based detectors were optimized by gradient descent exclusively on normal observations; labeled normal and anomalous samples were used separately for performance-guided model selection and anomaly-threshold calibration.

Operating-point selection and comparative performance assessment were based primarily on the $F_{1}$-score. The $F_{2}$-score was additionally reported as a complementary recall-oriented metric using the same model and scoring hyperparameters as the corresponding $F_{1}$-based configuration, but with a separately optimized decision threshold. Precision–recall-based evaluation is particularly informative in anomaly-detection settings with strongly imbalanced operational class distributions [11]. The metric definitions and threshold-selection procedure are detailed in Section 4.6 and Section 4.7.

The contribution of this study is a controlled assessment of these design factors within a common FM-spectrum reconstruction pipeline rather than the proposal of a new autoencoder family. Relative to our previous dataset-oriented work [7] and AE/CAE/VAE comparison [9], the present study assesses which pipeline components are most strongly associated with reconstruction-based anomaly-detection performance when gradient-based parameter updates are restricted to normal observations.

## 2. Related Work

### 2.1. Deep Reconstruction and One-Class Anomaly Detection

Deep anomaly-detection methods aim to learn representations that distinguish normal observations from samples that deviate from the underlying normal-data distribution. Existing approaches include reconstruction-based learning, one-class representation learning, self-supervised methods, and hybrid formulations [2]. Among these, reconstruction-based autoencoders are particularly attractive when representative anomalous training samples are unavailable because they can be trained exclusively on observations corresponding to normal operating conditions.

A fundamental limitation of conventional autoencoder-based anomaly detection is that high reconstruction capability does not necessarily translate into high detection capability. A sufficiently expressive model may generalize beyond the normal training distribution and accurately reconstruct anomalous input, thereby reducing the contrast between normal and abnormal reconstruction errors. Gong et al. addressed this limitation with the Memory-Augmented Autoencoder (MemAE), which constrains reconstruction through a memory containing prototypical representations of normal observations [12]. This approach illustrates an important principle for reconstruction-based anomaly detection: increasing model capacity alone is not necessarily beneficial unless the representation and reconstruction process remain sufficiently constrained by normality.

One-class approaches address the same problem from a different perspective. Deep SVDD learns a representation in which normal observations are mapped to a compact region of the latent space, and deviations from this region are used to identify anomalous samples [13]. Together, these approaches demonstrate that anomaly-detection performance depends not only on the underlying network architecture but also on how normality is represented and how deviations from that representation are quantified.

### 2.2. Anomaly Detection in the RF Spectrum

RF-spectrum anomaly detection introduces additional challenges because normal spectral behavior is itself dynamic. Signal activity, received power, propagation conditions, interference, and measurement noise can vary considerably over time, while anomalous events may differ in duration, bandwidth, and magnitude. Consequently, several RF anomaly-detection studies have investigated learned representations tailored to different forms of spectrum observations.

Feng et al. formulated spectrum anomaly detection as a novelty-detection problem and applied deep autoencoders to time–frequency representations [4]. Their experiments showed that architectural depth can substantially influence detection performance, highlighting the importance of model capacity in reconstruction-based RF analysis. Rajendran et al. proposed SAIFE, an adversarial-autoencoder-based framework operating on power spectral density (PSD) observations that combines unsupervised anomaly detection with localization and interpretable latent representations [3]. In contrast, Tschimben and Gifford investigated deep long short-term memory (LSTM) and LSTM autoencoders using IQ observations for spectrum-sharing and monitoring applications [5]. Because IQ measurements retain detailed amplitude and phase information, the resulting learning problem differs considerably from anomaly detection based on longer-term two-dimensional spectrum-monitoring representations.

More recently, Wang et al. proposed an adversarial-autoencoder-based method for anomalous radio-signal detection, further demonstrating the applicability of generative normality modeling to RF signals [6]. Beyond reconstruction-based detection, Milosheski et al. investigated self-supervised representation learning to group real-world wireless spectrum activity and demonstrated the feasibility of extracting informative representations without manually assigned activity labels [1]. This is particularly relevant to operational spectrum monitoring, where large-scale manual annotation is costly and requires domain expertise.

Tian et al. [14] proposed a VAE-based unsupervised spectrum anomaly-detection method for unauthorized frequency bands and introduced a percentile (PER) score designed to capture distributional changes in reconstruction errors. Their work further demonstrates that the formulation and aggregation of reconstruction residuals can represent an important design dimension in spectrum anomaly detection. In contrast to their unauthorized-band setting, the present study considers operational VHF Band II radio-broadcasting waterfall measurements and compares percentile scoring with several alternative residual-based scoring strategies within a broader pipeline-level evaluation.

Although these studies have established the feasibility of machine-learning-based RF anomaly detection, they differ substantially in their input representations, monitored frequency environments, anomaly definitions, and learning objectives. PSD vectors, IQ sequences, and time–frequency measurements retain different levels of spectral, temporal, and phase information and therefore cannot be expected to produce directly transferable architectural conclusions. In particular, comparatively limited evidence is available on the interaction between preprocessing, reconstruction capacity, and anomaly scoring for long-term FM waterfall measurements collected by operational spectrum-monitoring infrastructure.

Our previous study further showed that encoder-derived latent representations contain useful anomaly-related information that is not fully exploited by conventional reconstruction-error scoring, motivating the present focus on the complete reconstruction-based detection pipeline [9].

### 2.3. Visual Anomaly Detection and Reconstruction Scoring

Related methodological developments can also be found in visual anomaly detection, where learning from normal observations has received considerable attention. These approaches provide useful conceptual reference points because RF waterfall measurements can be represented as two-dimensional arrays, although their physical interpretation differs fundamentally from that of natural or industrial images.

Bergmann et al. investigated the use of structural similarity in autoencoder-based unsupervised defect segmentation and showed that the choice of reconstruction criterion can substantially affect anomaly-localization behavior [10]. The Structural Similarity Index (SSIM), originally introduced as an image-quality measure, evaluates local luminance, contrast, and structural relationships rather than relying exclusively on pointwise differences [15]. These findings demonstrate that the conversion of reconstruction differences into an anomaly measure is itself an important methodological decision.

Other visual anomaly-detection approaches reduce or eliminate the dependence on conventional pixel-level reconstruction. CutPaste uses artificially generated image transformations as pseudo-anomalies to learn discriminative representations in a self-supervised manner [16]. Discriminatively trained reconstruction embedding (DRAEM) similarly employs synthetically generated anomalies, but it combines reconstruction with a discriminatively trained anomaly-localization network [17]. PatchCore instead represents normality using a memory bank of local features extracted from a pre-trained network and determines anomaly scores from the distances between test features and representative normal patches [18]. EfficientAD combines the prediction of student–teacher characteristics with an autoencoder component while emphasizing both detection accuracy and computational efficiency [19].

These methods illustrate the broader transition in visual anomaly detection from simple global reconstruction error toward localized, feature-based, memory-based, or discriminatively learned anomaly measures. However, it cannot be assumed that their effectiveness transfers directly to RF waterfall measurements. Unlike conventional images, the axes and pixel intensities of a waterfall representation have explicit physical meanings: the horizontal and vertical dimensions encode frequency and time, respectively, while the pixel intensity represents measured signal level. Consequently, methods that rely on semantic features learned from natural-image datasets may require domain-specific validation before they can be considered effective for spectrum-monitoring data.

For reconstruction-based RF detection, the anomaly-score formulation remains particularly important. An autoencoder produces a spatially distributed residual between the input and its reconstruction, but a subsequent decision rule is required to convert this residual map into an observation-level anomaly score. Conventional mean squared error (MSE) performs global averaging, while SSIM incorporates local structural information [10,15]. Alternative strategies can instead emphasize the magnitude or distribution of localized residuals. The distinction is relevant because globally averaged reconstruction quality and anomaly separability represent related but fundamentally different optimization objectives.

### 2.4. Positioning of the Present Study

The literature therefore reveals two complementary limitations relevant to the present work. First, previous RF studies demonstrate the applicability of autoencoder-based anomaly detection but employ heterogeneous PSD, IQ, and time–frequency representations, making it difficult to derive general design recommendations for operational FM waterfall measurements [3,4,5,6]. Second, both general anomaly-detection research and visual anomaly-detection studies indicate that detection performance is determined not only by neural-network architecture but also by the representation of normality and the formulation of the anomaly score [10,12,13,15].

A complementary task-oriented perspective has also emerged in machine-learning-based sensing and monitoring research. Mahmood et al. organize learning-based structural health monitoring by linking sensing configurations, data characteristics, and downstream monitoring tasks [20]. Although the sensing modality differs from RF spectrum monitoring, this systems-level perspective reinforces the importance of aligning preprocessing, data representation, learning, anomaly scoring, and decision-making stages with the intended monitoring task.

Taken together, previous studies leave open how preprocessing, reconstruction capacity, and residual-based anomaly scoring interact in operational FM waterfall anomaly detection. The present study addresses this gap within a common reconstruction-based experimental framework, with particular emphasis on whether localized residual aggregation can improve anomaly discrimination compared with globally aggregated reconstruction measures.

## 3. Dataset and Spectrum Image Generation

### 3.1. Source Measurement Dataset

The measurement data used in this study originate from the publicly available *Hungarian FM Spectrum Measurements Dataset*, released as a versioned research dataset on Zenodo [8]. The dataset contains real-world radio-frequency measurements acquired in Hungary and includes observations of the 87.5–108 MHz very high frequency (VHF) FM sound-broadcasting band. The corresponding measurement methodology, acquisition procedure, and dataset construction process are described in our previous data-oriented work [7].

Original measurements are stored in tabular CSV format. The consecutive rows correspond to measurement instants, while the individual frequency bins are represented by separate columns [7]. This representation preserves the numerical measurement values and is directly suitable for conventional signal-processing workflows. However, the anomaly-detection models considered in the present study operate on two-dimensional time–frequency representations. A dedicated preprocessing pipeline was therefore applied to standardize the temporal and frequency resolutions and subsequently transform the measurement matrices into spectrum images.

### 3.2. Temporal and Frequency Standardization

Measurements originating from different acquisitions can have different native temporal and frequency resolutions. Before spectrum-image generation, both dimensions were therefore standardized to obtain a consistent physical interpretation of the resulting image structures across measurement files, as follows.

Let $\Delta f_{\mathrm{native}}$ denote the native frequency spacing and $\Delta t_{\mathrm{native}}$ the native temporal sampling interval. The frequency spacing is determined from two consecutive frequency values in the CSV header, whereas the temporal interval is calculated from the timestamps of consecutive measurements. The target resolutions used in the preprocessing procedure are $\Delta f_{\mathrm{target}}=5\;\mathrm{kHz}$, $\Delta t_{\mathrm{target}}=400\;s$. Rather than interpolating the original measurements, the implemented preprocessing procedure performs index-based subsampling. The approximate index strides in the frequency and temporal dimensions are determined as

$$k_{f}=\mathrm{round}\left(\frac{\Delta f_{\mathrm{target}}}{\Delta f_{\mathrm{native}}}\right),\;k_{t}=\mathrm{round}\left(\frac{\Delta t_{\mathrm{target}}}{\Delta t_{\mathrm{native}}}\right),$$

where every $k_{f}$-th frequency bin and every $k_{t}$-th temporal observation are retained. This approach preserves measured samples and avoids the generation of artificial spectral values through interpolation. Because integer index strides are used, 5 kHz and 400 s represent target resolutions; the exact resulting sampling intervals can differ slightly when the ratio between the target and native resolutions is not an integer.

The preprocessing procedure allows for downsampling only. If the native frequency resolution is coarser than the specified 5 kHz target resolution, the procedure is terminated instead of artificially increasing the spectral resolution. The same principle is applied in the temporal dimension. Missing numerical entries encountered during CSV loading were replaced by zero to ensure a complete numerical matrix for subsequent processing. A fixed replacement was preferred over interpolation to avoid introducing artificial temporal or spectral structures into the measurement data.

Standardizing both dimensions ensures that the spatial structures in the generated images have approximately consistent physical meanings. Consequently, a structure spanning the same number of horizontal pixels corresponds to approximately the same frequency bandwidth, while a structure spanning the same number of vertical pixels represents approximately the same temporal duration across the processed measurements.

### 3.3. Generation of Spectrum Images

Following temporal and frequency standardization, the timestamp column was removed and the remaining numerical matrix was interpreted as a two-dimensional spectrum representation. The matrix columns corresponded to frequency bins, while consecutive rows represented temporal observations. The standardized measurement matrix was divided into non-overlapping tiles of size 640×640 pixels, while incomplete tiles at the temporal or frequency boundaries were discarded rather than padded or resized, ensuring identical spatial dimensions for all generated spectrum images.

**Figure 1:**
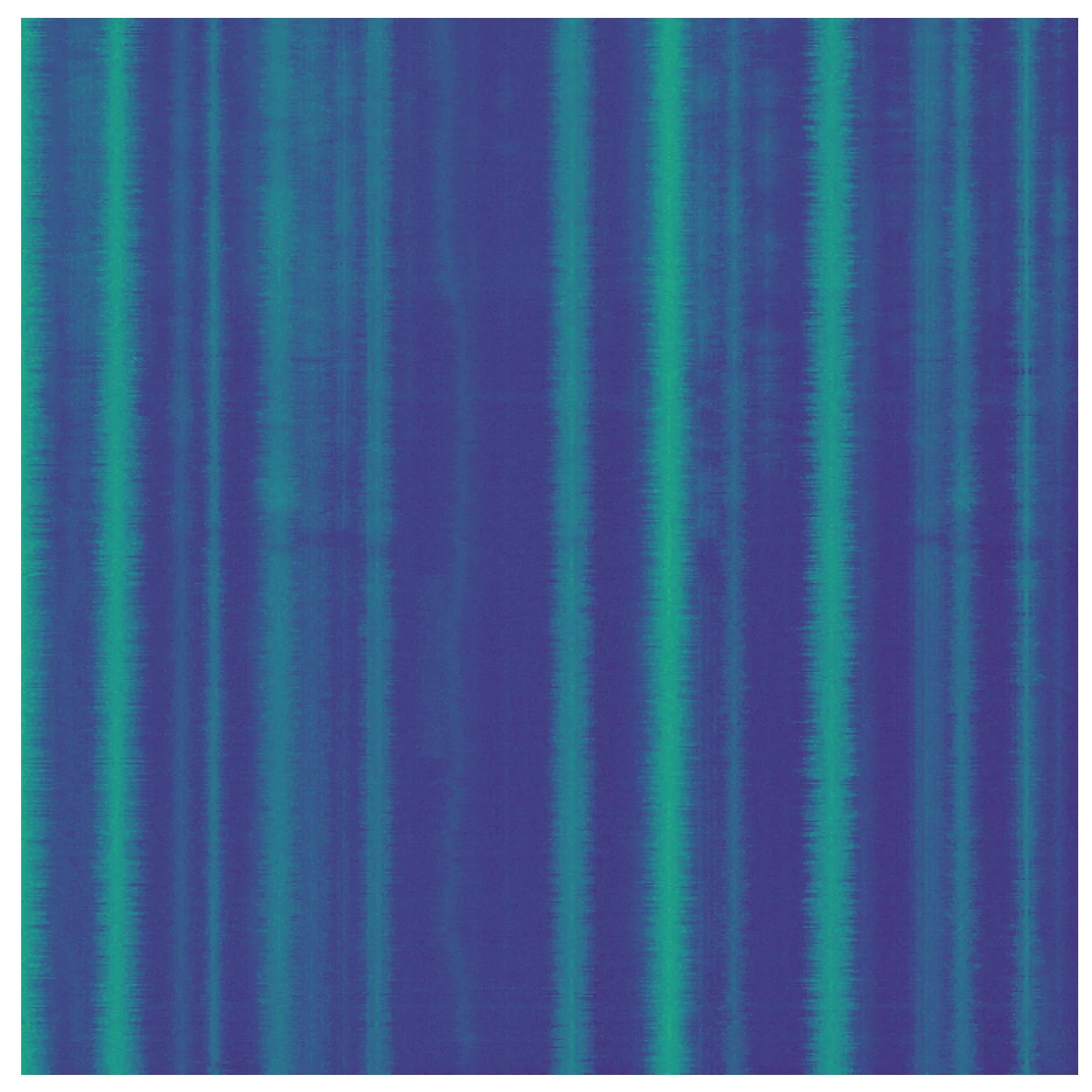
Example of a VHF Band II radio-broadcasting spectrum image generated from the original CSV measurement data after temporal and frequency standardization. The horizontal axis corresponds to frequency, the vertical axis represents time, and the color scale encodes the measured spectral level using identical visualization limits for all generated images.

Each resulting tile was converted into a PNG spectrum image using an identical visualization procedure. A fixed measurement range of −20 to 100 dBμV/m was applied to every generated image, and the jet color map was used throughout the image-generation process. The use of global visualization limits prevented sample-specific re-scaling during spectrum-image generation and ensured that a given measured level was initially represented by the same color across the generated RGB images. Subsequent model-specific intensity normalization was applied separately during preprocessing, as described in Section 4.2. An example of a spectrum image generated using this procedure is shown in Figure 1.

No axes, tick labels, titles, or other graphical annotations were included in the model input. Thus, only information derived from the measured spectrum was provided to the anomaly-detection models. Subsequent model-specific resizing and preprocessing are described in Section 4.2.

### 3.4. Normal and Anomalous Spectrum Observations

The generated waterfall images exhibit structured but heterogeneous spectral activity. Under normal operating conditions, FM broadcasting signals appear as persistent spectral structures whose center frequencies and characteristic spectral patterns remain relatively stable over time. However, their measured intensity can vary due to propagation conditions, monitoring location, background noise, and other environmental measurement effects. Normality therefore cannot be represented by a single fixed spectrum pattern.

For the purposes of this study, anomalous observations correspond to deviations from the expected temporal or spectral behavior of the monitored transmissions. Three principal types of abnormal behavior are represented in the development data:

- transmission interruption, in which an otherwise persistent broadcast signal temporarily disappears;
- loss of modulation, in which the characteristic modulated spectral structure is lost while a carrier-related component may remain observable; and
- abnormal frequency displacement, in which the signal energy deviates from its expected spectral location.

The spatial and temporal extent of these events varied considerably. In particular, some anomalous regions occupied only a small fraction of the complete waterfall image, creating an important challenge for reconstruction-based anomaly scoring in the presence of spatially distributed measurement noise.

The composition of the location-separated training and development subsets is summarized in Table 1. Model training used exclusively normal observations, whereas both normal and anomalous observations from different monitoring locations were included in the development subset.

The training and development subsets were constructed based on the origin of the measurements rather than using random image-level partitioning. Measurements assigned to the training and development subsets were acquired at different monitoring locations, thus preventing spectrum images originating from the same monitoring location from appearing in both subsets. All measurements were acquired using the same measurement instrument, while the native temporal and frequency resolutions could differ between acquisitions. Before spectrum-image generation, these differences were standardized using the procedure described in Section 3.2, with target frequency and temporal spacings of 5 kHz and 400 s, respectively. Consequently, the 640×640 source tiles used for image generation had approximately consistent physical sampling characteristics at the different measurement locations.

An additional independently held-out test subset was constructed for generalization assessment. This subset contained 37 normal and 53 anomalous observations, resulting in 90 held-out spectrum images. The held-out observations were excluded from all stages of model development, including gradient-based optimization, architecture selection, checkpoint selection, reconstruction-scoring hyperparameter selection, and decision-threshold calibration. Consequently, test-set labels were used only for the final calculation of evaluation metrics after all model and operating-point choices had been frozen.

The approximately balanced composition of the development subset was used to allow for controlled comparison between detection configurations and should not be interpreted as representative of the prevalence of anomalous events in operational spectrum monitoring, where anomalies are substantially less frequent.

**Figure 2:**
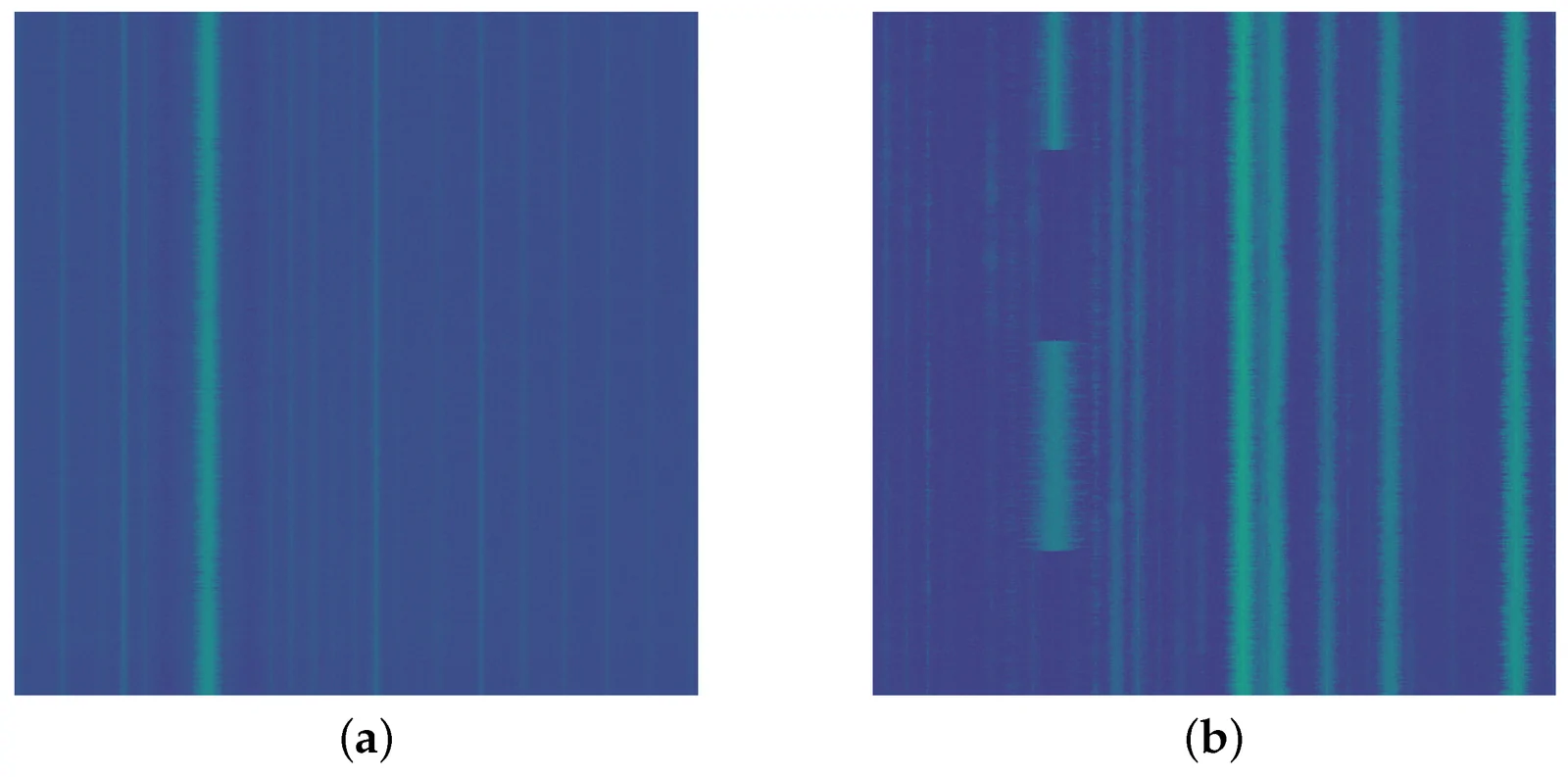
Representative examples of normal and anomalous VHF Band II radio-broadcasting spectrum observations: (**a**) Normal spectrum image exhibiting temporally persistent broadcast signals without evident abnormal behavior. (**b**) Anomalous spectrum image containing a deviation from the expected temporal or spectral structure of one or more transmissions. The examples illustrate that an anomalous region may occupy only a limited fraction of the complete waterfall representation.

Normal/anomalous labels were assigned manually by visually inspecting the generated spectrum images and identifying deviations from the expected temporal and spectral behavior of FM transmissions. The detailed annotation procedure and anomaly definitions are provided in [7]. Representative examples of normal and anomalous spectrum observations are shown in Figure 2.

## 4. Materials and Methods

### 4.1. Overall Anomaly Detection Framework

**Figure 3:**
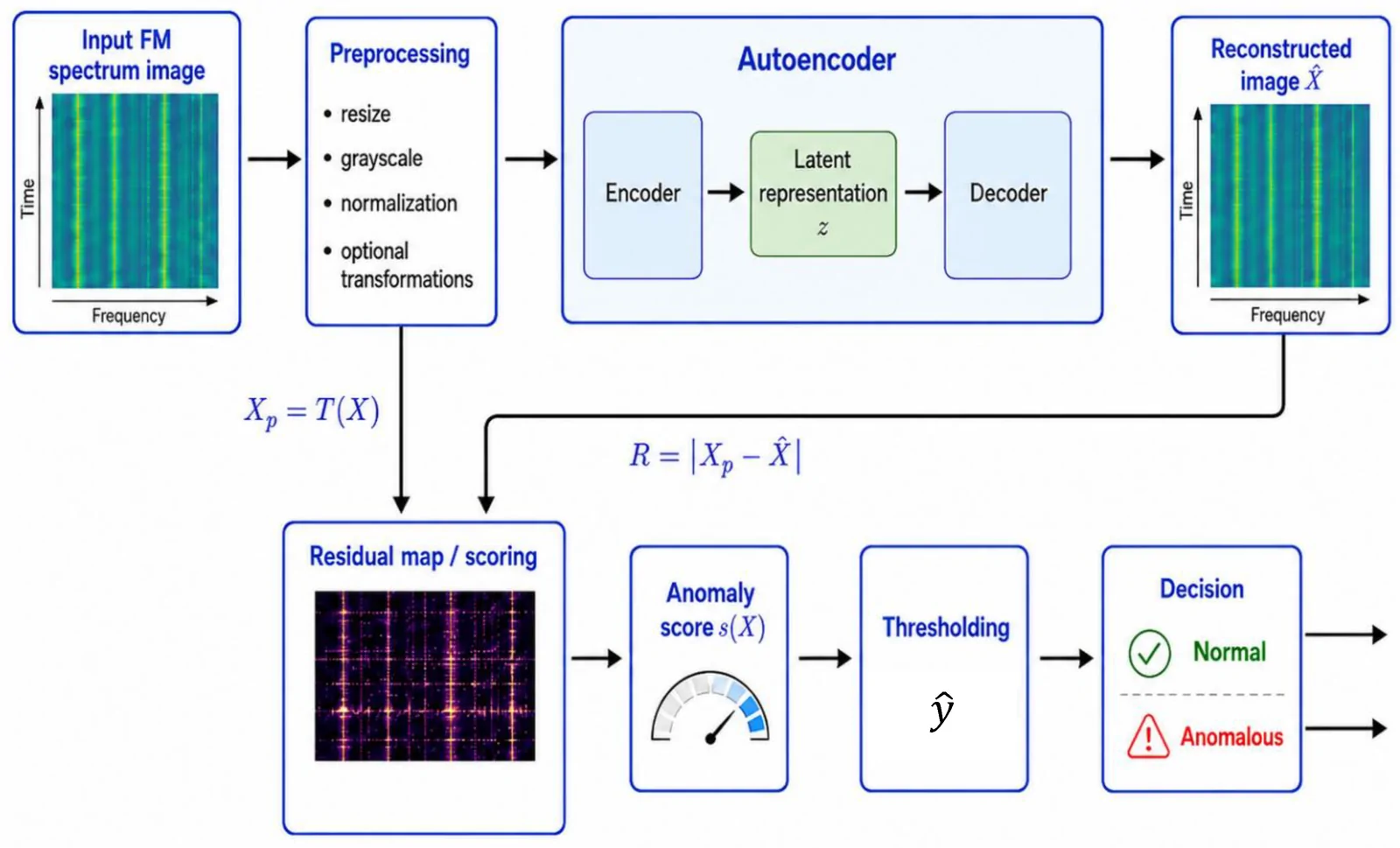
An overview of the proposed reconstruction-based VHF Band II broadcasting-spectrum anomaly-detection framework. The input spectrum image first undergoes a selected preprocessing step and is then reconstructed by an autoencoder trained exclusively on normal observations. An anomaly score is generated from the difference between the input and the reconstruction, and this score is compared to a decision threshold to classify the data as normal or anomalous.

The proposed anomaly-detection framework is based on autoencoder reconstruction learning and is organized as a sequential pipeline consisting of input preprocessing, representation learning, reconstruction, residual-based scoring, and anomaly decision. Rather than considering the autoencoder as an isolated model, the complete processing chain is evaluated since each stage can influence the separation between normal and anomalous spectrum observations. The general framework is illustrated in Figure 3.

Let *X* denote an input FM spectrum image and let T(·) represent the selected preprocessing transformation. The processed observation is therefore given by $X_{p}=T\left(X\right)$, where $X_{p}$ denotes the input representation supplied to the reconstruction model.

Depending on the investigated configuration, the preprocessing stage may include grayscale conversion, intensity normalization, resolution reduction, noise-suppressing intensity transformations, edge-enhanced representations, or spectrum-specific rearrangements of the input. Individual transformations are described in detail in Section 4.2. Keeping preprocessing as an explicit component of the framework makes it possible to evaluate whether the detectability of spectral anomalies is determined solely by the neural architecture or is also strongly affected by the representation presented to the model.

The reconstruction model consists of an encoder E(·) and a decoder D(·). The encoder transforms the preprocessed spectrum image into a latent representation $z=E(X_{p})$, while the decoder reconstructs the input from this representation,

$$\hat{X}=D\left(z\right)=D\left(E\left(X_{p}\right)\right),$$

where $\hat{X}$ denotes the reconstructed spectrum image.

The autoencoders are trained using only normal spectrum observations. Consequently, no anomalous examples are required to optimize network parameters. The reconstruction objective encourages the model to learn the characteristic spectral structures that occur during normal operation. During inference, both normal and anomalous observations are processed by the same trained model. The underlying assumption is not that every anomalous observation necessarily produces a uniformly poor reconstruction, but rather that deviations from the learned normal spectrum structure can produce reconstruction discrepancies that are distinguishable through an appropriate anomaly-scoring strategy.

After reconstruction, the discrepancy between the preprocessed input and its reconstruction is converted into an anomaly score using the residual-based scoring strategies described in Section 4.4. Finally, the anomaly score is converted into a binary decision using the thresholding procedure described in Section 4.6.

A central aspect of the experimental design is that the individual components of this pipeline are varied while the overall anomaly-detection principle remains unchanged. The study therefore evaluated the effects of input representation, network depth, latent-space dimensionality, encoder–decoder structure, and reconstruction-scoring strategy within a common framework. Symmetric convolutional autoencoders served as the principal reference architecture, while asymmetric, stacked, and variational configurations were investigated as alternative mechanisms for controlling representation and reconstruction capacity. This formulation supported a comparative assessment of which stages of the reconstruction-based pipeline are most strongly associated with anomaly-detection performance in noisy FM spectrum measurements.

### 4.2. Input Preprocessing and Spectrum Representations

The 640×640 RGB spectrum images generated in Section 3 were not used directly as autoencoder inputs. Instead, several preprocessing and alternative representation strategies were evaluated to reduce dimensionality and nuisance variability while preserving structures relevant to anomaly detection.

#### 4.2.1. Baseline Preprocessing

The baseline preprocessing procedure consisted of spatial downsampling, grayscale conversion, and intensity normalization. We let $X\in R^{640\times 640\times 3}$ denote an original RGB spectrum image. The image was first resized from 640×640 to 128×128 pixels,

$$X_{r}=R\left(X\right),$$

where R(·) denoted the resizing operation. The reduction in spatial resolution considerably decreased the computational and memory requirements of the subsequent convolutional autoencoders and also reduced the influence of fine-scale variations in the original representation.

The resized RGB image was subsequently converted to a single grayscale channel, $X_{g}=G\left(X_{r}\right)$, where G(·) denoted grayscale conversion. The RGB channels of the original spectrum images originated from the visualization of a single measured spectral-level quantity and therefore did not represent three independent physical variables. Grayscale conversion reduced the dimensionality of this redundant visual representation before reconstruction learning.

Finally, image-wise min–max normalization was applied independently to each grayscale image:

$$X_{n}=\frac{X_{g}-\min\left(X_{g}\right)}{\max\left(X_{g}\right)-\min\left(X_{g}\right)+\varepsilon }\;,$$

where $\varepsilon =10^{-8}$ was a small numerical constant preventing division by zero and $X_{n}\in {\left[0,\;1\right]}^{128\times 128}$ denoted the normalized model input. Unlike the fixed −20 to 100 dBμV/m visualization range used during spectrum-image generation, this operation performed sample-specific intensity normalization after RGB-to-grayscale conversion. Consequently, the absolute intensity scale was not preserved in the final model input; instead, the relative intensity distribution within each observation was emphasized. This transformation was intentionally used to reduce variations in overall received signal level between measurements acquired at different monitoring locations while preserving the relative time–frequency structures relevant to anomaly detection.

**Figure 4:**
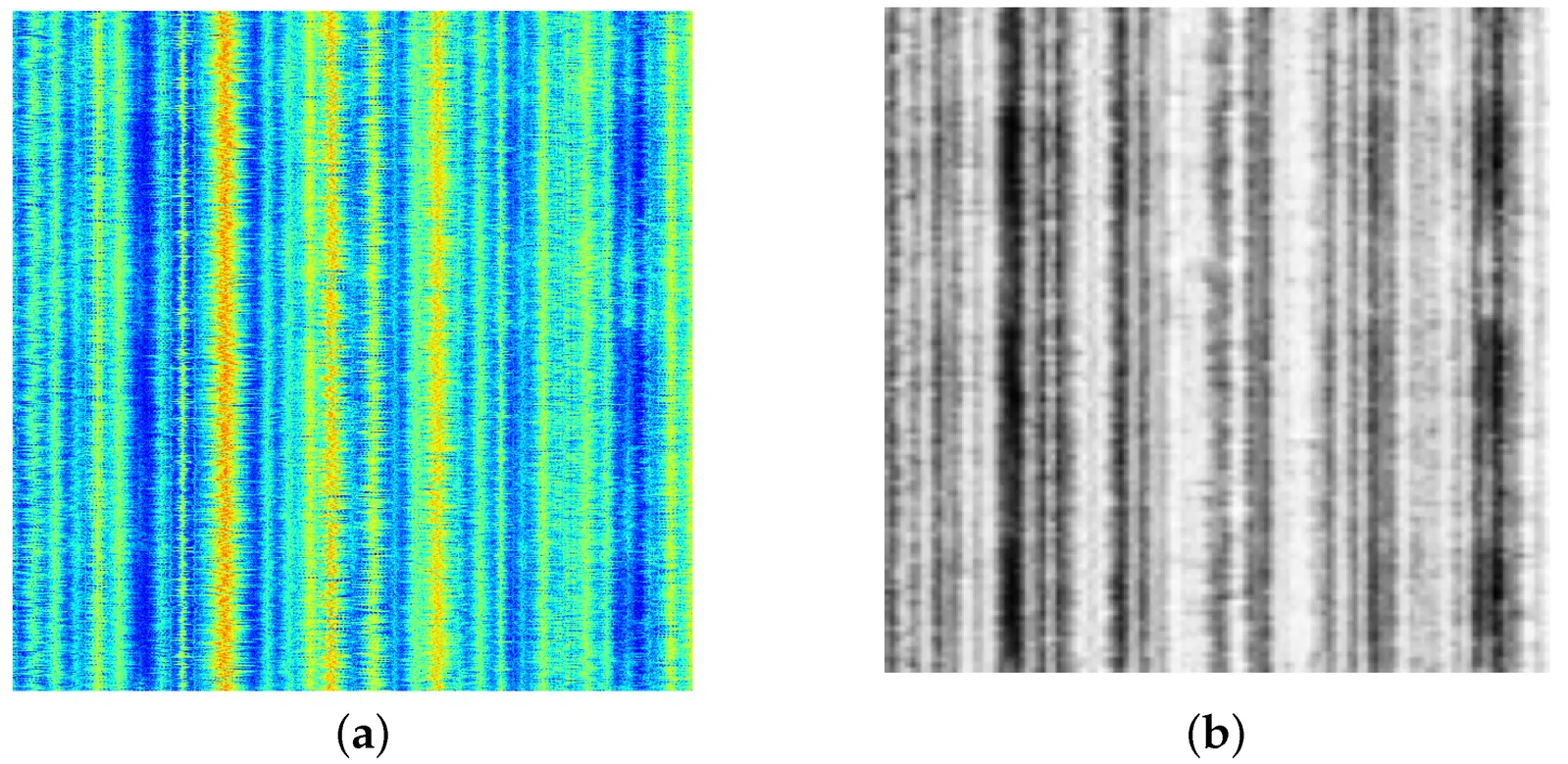
Effect of the baseline preprocessing procedure on an FM spectrum observation: (**a**) original 640 × 640 RGB waterfall image; (**b**) corresponding 128 × 128 grayscale image after resizing, grayscale conversion, and min–max normalization to the [0, 1] interval. The horizontal dimension represents frequency and the vertical dimension represents time.

The complete baseline preprocessing transformation can therefore be expressed as

$$X_{n}=N\left(G\left(R(X)\right)\right),$$

where R(·), G(·), and N(·) denote resizing, grayscale conversion, and min–max normalization, respectively. Unless otherwise stated, $X_{n}$ was used as the reference representation in the subsequent experiments. The effect of the complete baseline preprocessing procedure on a representative spectrum image is illustrated in Figure 4.

#### 4.2.2. Intensity-Domain Transformations

In addition to the baseline representation, two intensity-domain transformations were evaluated to reduce the contribution of weak background fluctuations.

The first transformation applied hard intensity masking. Given a predefined masking threshold *m*, the transformed image was defined pixel-wise as

$$X_{m}\left(i,j\right)=\left\{\begin{array}{cc} 0, & X_{n}\left(i,j\right)<m,\\ X_{n}\left(i,j\right), & X_{n}\left(i,j\right)\geq m, \end{array}\right.$$

where $X_{n}\left(i,j\right)$ denoted the normalized intensity in the spatial position (i,j). The motivation for masking was to suppress low-intensity components that might predominantly represent weak background fluctuations or measurement noise. This transformation increased the relative prominence of stronger spectral structures, although the use of a hard threshold might also remove weak but potentially informative components and introduced an abrupt transition at *m*. In the evaluated masking configuration, the threshold was set to m=0.1.

**Figure 5:**
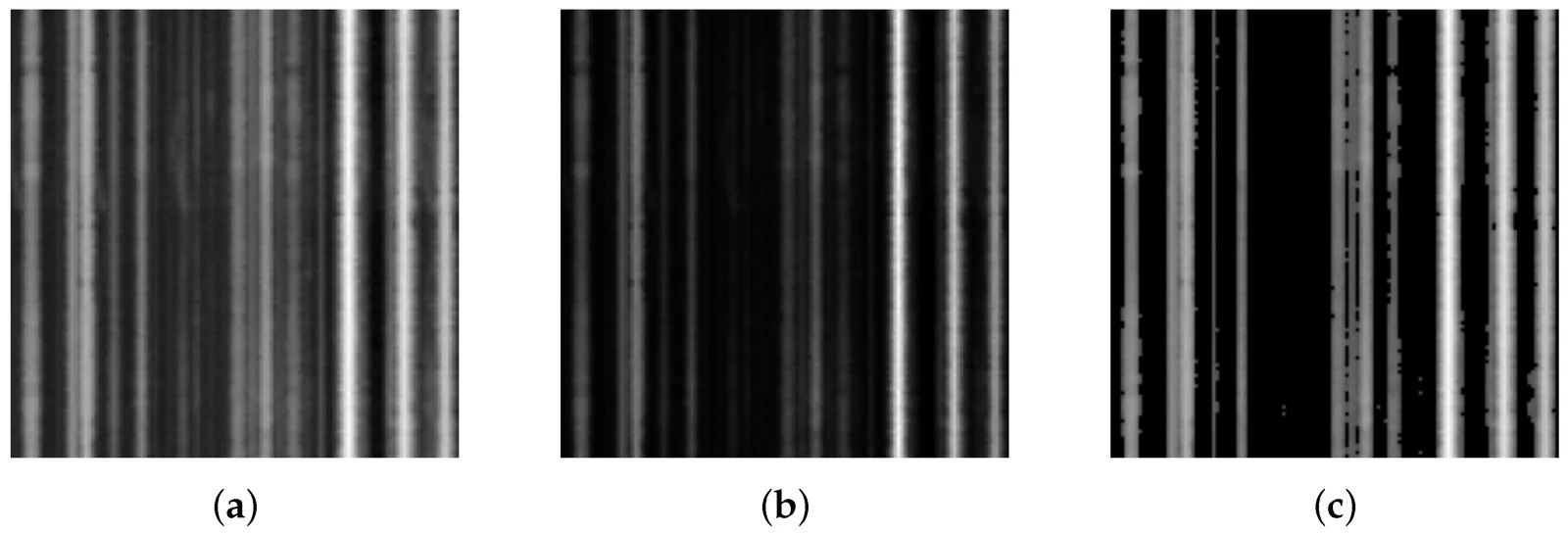
Comparison of intensity-domain preprocessing strategies: (**a**) Baseline grayscale and normalized spectrum representation. (**b**) Power-transformed representation, in which low-intensity components are nonlinearly attenuated. (**c**) Masked representation, in which pixels below a predefined threshold are set to zero.

As a smoother alternative, a nonlinear power transformation was applied to the normalized image:

$$X_{p}\left(i,j\right)={\left[X_{n}\left(i,j\right)\right]}^{\gamma },\;\gamma >1,$$

where γ controlled the strength of the nonlinear suppression. Because $X_{n}\left(i,j\right)\in \left[0,\;1\right]$, exponentiation with γ>1 attenuated low-intensity values more strongly than values close to unity. Consequently, weak background components were suppressed while stronger spectral structures retained a comparatively larger contribution. Unlike hard masking, the power transformation preserves continuous intensity transitions. In the evaluated power-transformation configuration, the exponent was set to γ=3. The visual effects of the baseline, power-transformed, and masked representations are compared in Figure 5.

In addition to modifying pixel intensities, an edge-enhanced representation was investigated because several relevant anomaly types correspond to abrupt changes in the temporal or spectral structure of an FM transmission. For example, a transmission interruption produces a discontinuity in a signal that is otherwise persistent over time. Edge information was extracted from the baseline representation using the Sobel operator. The two convolution kernels were

$$K_{x}=\left[\begin{array}{ccc} -1 & 0 & 1\\ -2 & 0 & 2\\ -1 & 0 & 1 \end{array}\right],\;K_{y}=\left[\begin{array}{ccc} -1 & -2 & -1\\ 0 & 0 & 0\\ 1 & 2 & 1 \end{array}\right].$$

and the resulting gradient magnitude was calculated as

$$G_{x}=X_{n}*K_{x},\;G_{y}=X_{n}*K_{y},$$

$$G\left(i,j\right)=\sqrt{G_{x}{\left(i,j\right)}^{2}+G_{y}{\left(i,j\right)}^{2}}.$$

**Figure 6:**
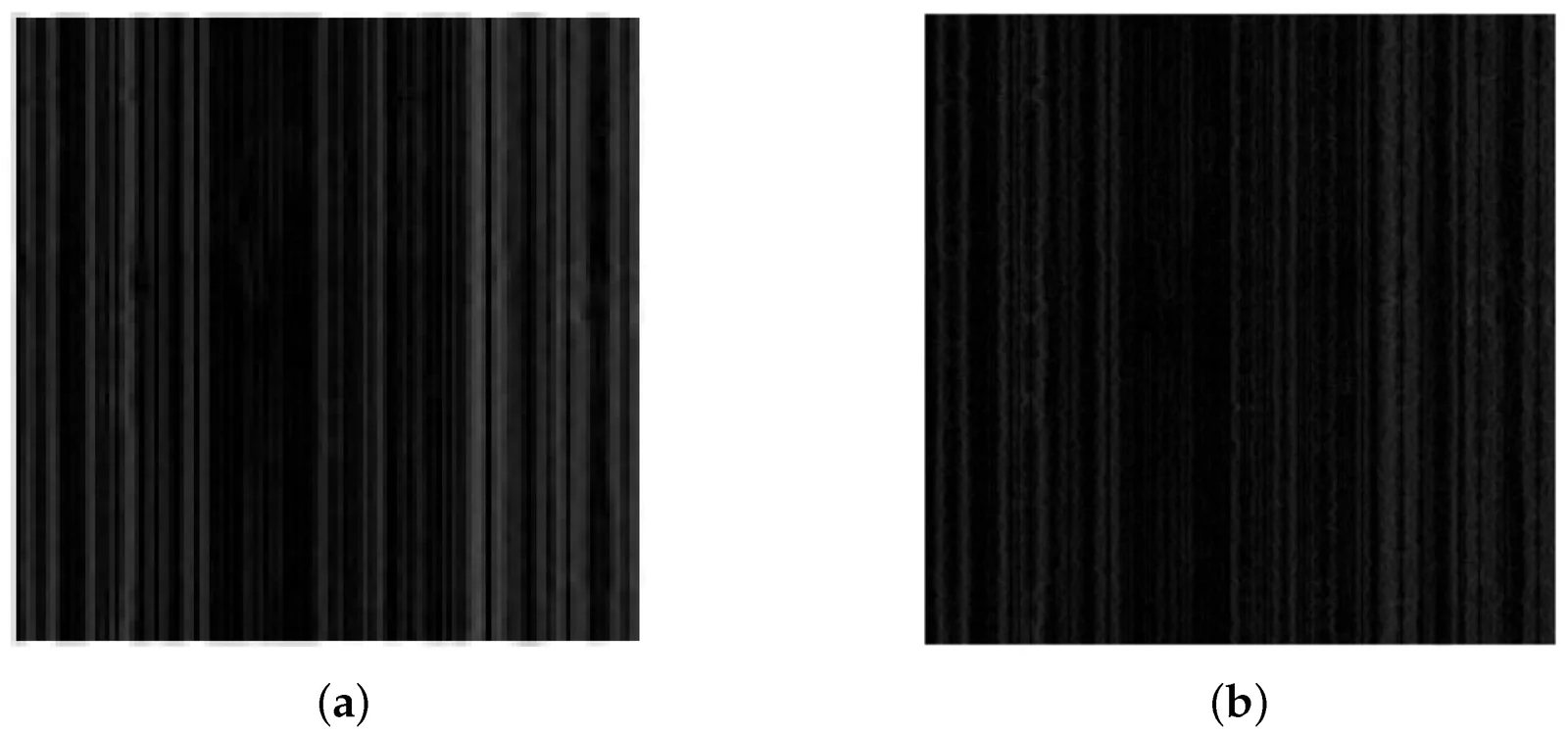
Sobel edge maps used to construct the edge-augmented two-channel representations: (**a**) 128×128 input resolution and (**b**) 320×320 input resolution.

The edge map was not used as a standalone model input. Instead, it was concatenated with the corresponding intensity representation, resulting in a two-channel input containing the spectrum intensity and its Sobel gradient magnitude. Subsequently, channel-wise percentile normalization using the 1st and 99th percentiles as the lower and upper normalization bounds, respectively, was applied before reconstruction. Since frequency corresponded to the horizontal dimension and time to the vertical dimension, the two gradient components emphasized transitions along different physical dimensions of the measurement. The motivation for this representation was to suppress slowly varying intensity information while increasing the prominence of abrupt temporal and spectral boundaries. To assess the trade-off between preserved spatial detail and noise sensitivity, edge-augmented representation was evaluated at both input resolutions 128×128and320×320. Representative Sobel edge maps for the two resolutions are shown in Figure 6.

**Figure 7:**
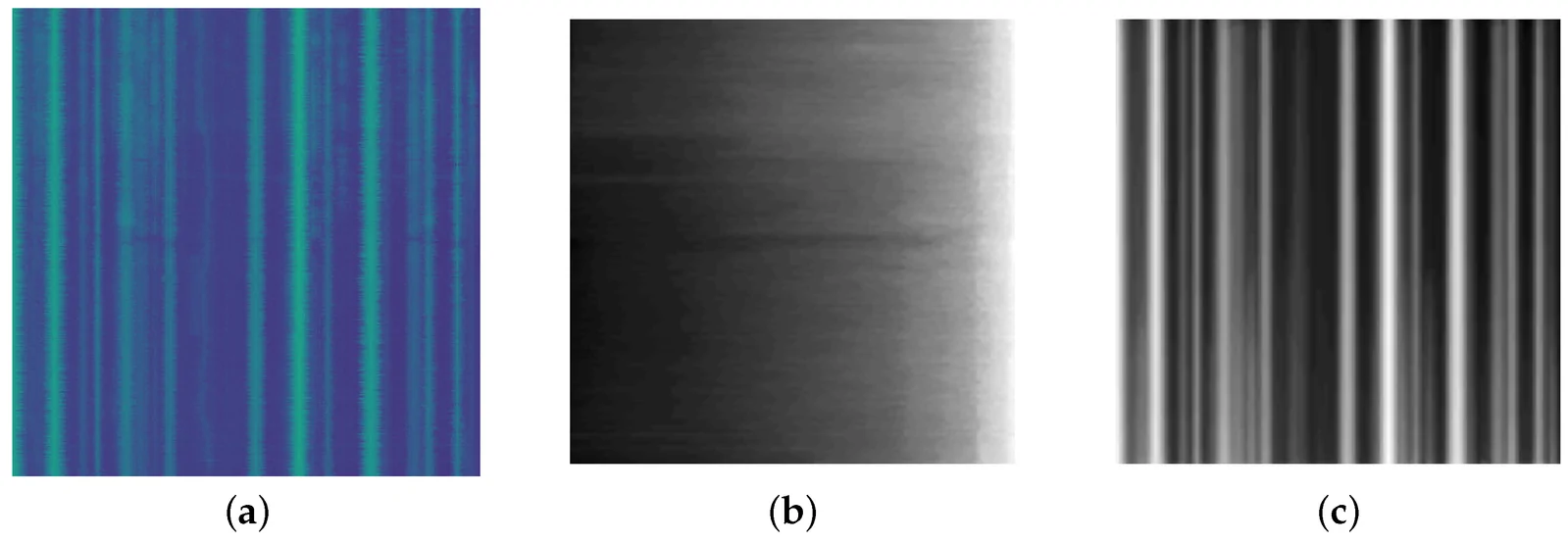
Axis-wise pixel representations derived from the baseline-preprocessed FM spectrum image. (**a**) Original preprocessed representation. (**b**) Pixel intensities sorted along the frequency dimension. (**c**) Pixel intensities sorted along the temporal dimension. The two transformations emphasize complementary characteristics related to spectral occupancy and temporal persistence.

Finally, a spectrum-specific transformation based on axis-wise pixel sorting was introduced to expose characteristics that might be difficult to identify directly from the original spatial arrangement. We let $X_{b}\in {\left[0,\;1\right]}^{H\times W}$ where W represented the frequency dimension and H the temporal dimension. Two alternative representations were generated by independently sorting the pixel intensities along the two principal axes. Sorting along the frequency dimension was defined as $X_{f}\left(i,:\right)=sort\left(X_{b}\left(i,:\right)\right)$ where the values of each temporal observation were independently ordered according to intensity. Sorting along the temporal dimension was expressed similarly as $X_{t}\left(:,j\right)=sort\left(X_{b}\left(:,j\right)\right)$ where the intensity observations associated with each frequency position were independently ordered over time. The purpose of these transformations was not to preserve the original spatial organization but to expose differences in intensity distributions and signal persistence. After sorting, interruptions in otherwise persistent transmissions may produce characteristic changes in the resulting intensity profile, while variations associated with frequency displacement can alter the shape of the complementary representation. Because the two sorting directions emphasized different properties of the spectrum data, they were processed by separate autoencoder models rather than being combined into a single input. The resulting frequency- and time-axis-sorted representations are illustrated in Figure 7.

In general, the preprocessing strategies investigated correspond to different assumptions regarding the information most relevant to reconstruction-based spectrum anomaly detection. The baseline representation preserves the overall time–frequency organization while reducing station-dependent intensity variation; masking and power transformations alter the contribution of weak spectral components; edge enhancement emphasizes abrupt structural transitions; and axis-wise sorting deliberately reorganizes the data to expose signal-persistence characteristics. Subsequently, these alternative representations were evaluated using the autoencoder architectures described in Section 4.3 to determine how the input representation influences the quality of the reconstruction and the performance of anomaly detection.

### 4.3. Autoencoder Architectures

A set of convolutional autoencoder architectures was investigated to determine how reconstruction capacity, network depth, latent-space dimensionality, and encoder–decoder symmetry influence anomaly detectability. All the architectures followed the general reconstruction framework introduced in Section 4.1: an encoder $E_{\theta_{e}}$ mapped the preprocessed spectrum representation $X_{p}$ to a latent representation *z*, while a decoder $D_{\theta_{d}}$ reconstructed the corresponding input,

$$z=E_{\theta_{e}}\left(X_{p}\right),\;\hat{X}=D_{\theta_{d}}\left(z\right)$$

Because reconstruction capacity was a central design variable in this study, the evaluated architectures were configured to compare different degrees of compression and decoder expressiveness. The aim was to preserve sufficient capacity for normal spectral structure while avoiding unnecessarily unconstrained reconstruction.

The initial reference architecture was a convolutional symmetric autoencoder consisting of four convolutional stages in the encoder, a fully connected bottleneck, and four corresponding decoding stages. Each encoder stage applied a 3×3 convolution with unit padding, preserving spatial dimensions before downsampling. The number of feature maps increased from 16 in the first layer to 32, 64, and 128 in the subsequent stages. Spatial downsampling was performed using max pooling rather than strided convolution, resulting in the sequence

128×128⇒64×64⇒32×32⇒16×16⇒8×8

**Figure 8:**
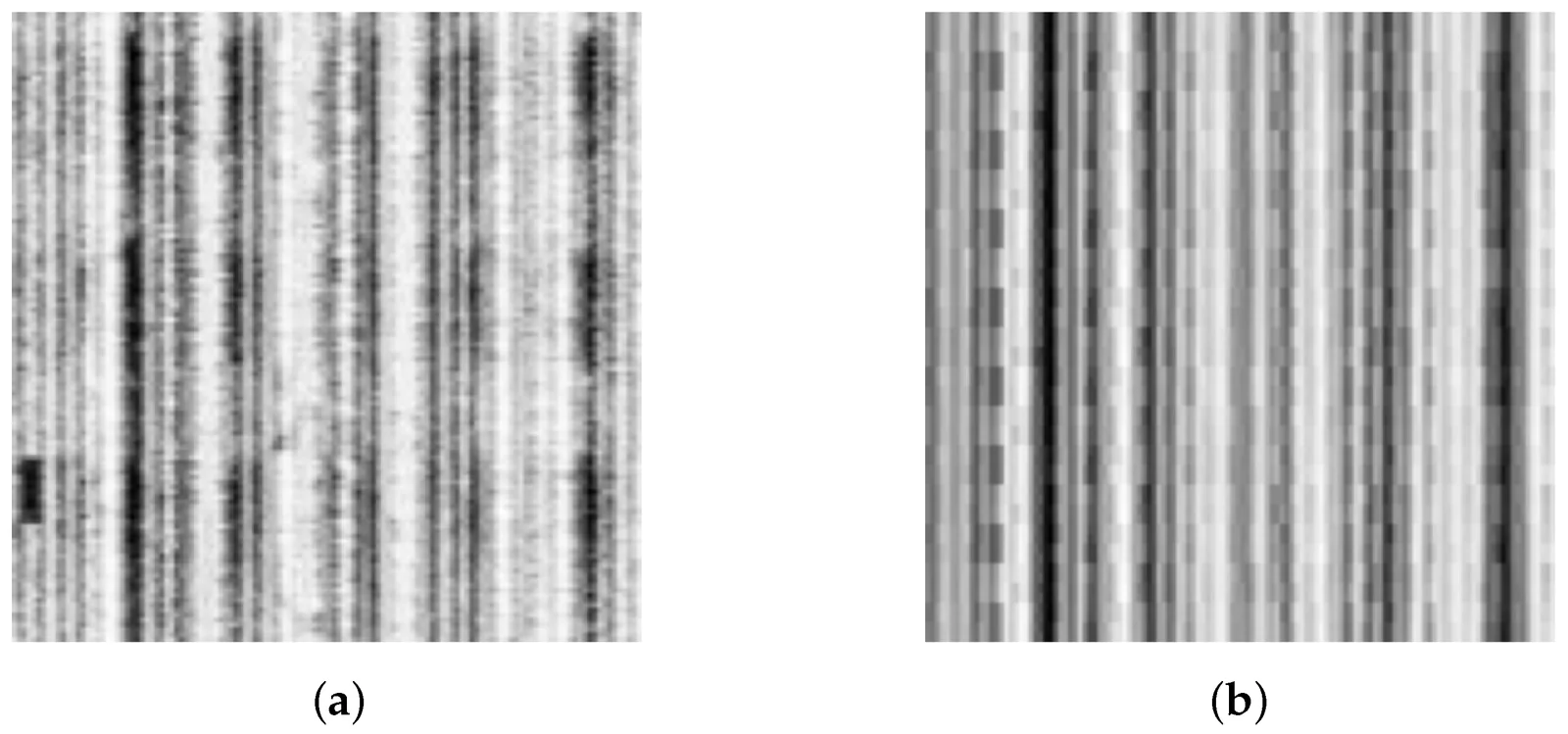
Example reconstruction obtained using the symmetric convolutional autoencoder with grayscale and normalized spectrum input: (**a**) preprocessed input image and (**b**) corresponding reconstruction. The model reconstructs the dominant normal spectral structures while suppressing part of the fine-grained variability. Weak checkerboard artifacts introduced by transposed convolution may be visible in the reconstructed representation.

Thus, for the grayscale input used by the principal models, the final convolutional representation had dimensions 128×8×8. This representation was flattened and projected by a fully connected layer into the latent space. Rectified linear unit (ReLU) activation functions were used within the reference encoder, together with a Dropout2d with a probability of p=0.1 to reduce overfitting. The decoder performed the inverse transformation and restored the spatial dimensions using transposed convolutions. Transposed convolutions were retained in the evaluated implementation for spatial upsampling in the decoder, although they may introduce weak checkerboard patterns in reconstructed images. The original RGB baseline and the principal preprocessed model shared this general architecture but differed in input dimensionality and bottleneck capacity. The RGB baseline operated on three-channel inputs and used a strongly constrained latent vector of $d_{z}=8$. After introducing the grayscale and normalization procedure described in Section 4.2, the first and final convolutional layers were adapted to single-channel input and output, respectively, and the latent dimensionality was increased to $d_{z}=32$. The latter configuration served as the principal symmetric CAE reference architecture throughout the subsequent experiments. An example of the reconstruction produced by the symmetric CAE is shown in Figure 8.

Network depth and input resolution were treated as explicit design variables. Both 320×320 and 128×128 inputs were evaluated; for the 128×128 setting, three-, four-, and five-stage convolutional architectures were tested together with variants that modified or removed the fully connected bottleneck. These configurations were used to assess the trade-off between feature-extraction capacity and constrained reconstruction; comparative results are reported in Section 5.1.

An asymmetric convolutional autoencoder was investigated to determine whether feature extraction and reconstruction capacity should be controlled independently. In a conventional symmetric architecture, increasing the depth of the encoder typically produces a corresponding increase in the decoder capacity. Such a decoder may become sufficiently expressive to reconstruct not only normal structures but also previously unseen deviations. The asymmetric design therefore uses a deeper encoder together with a shallower decoder. Rather than imposing the primary information constraint through an extremely narrow bottleneck, the reconstruction capacity is restricted mainly on the decoder side.

**Figure 9:**
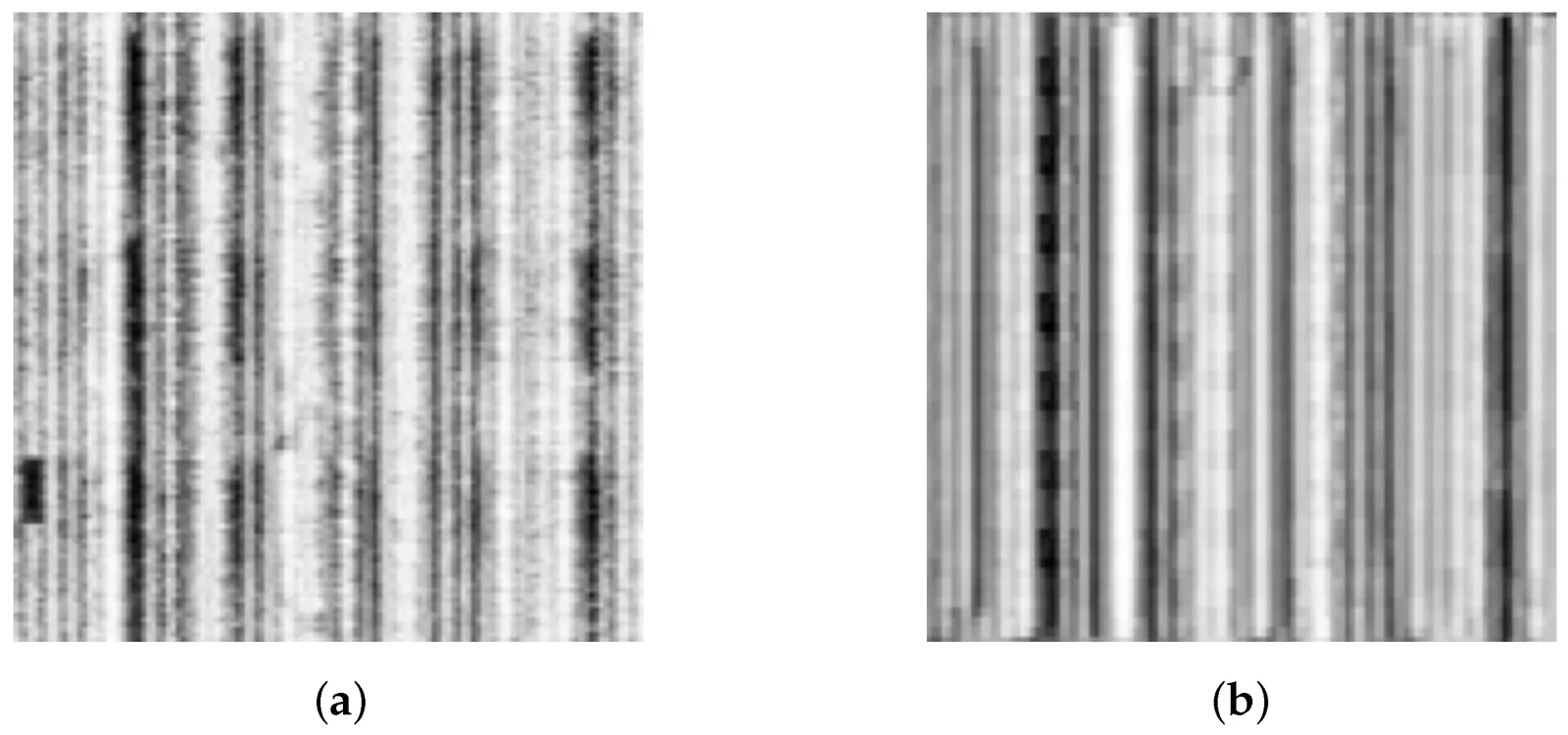
Example reconstruction produced by the asymmetric convolutional autoencoder: (**a**) preprocessed input image and (**b**) corresponding reconstruction. The encoder contains one additional processing stage relative to the decoder, thereby separating feature-extraction capacity from reconstruction capacity.

The asymmetric configuration used in the main comparison consisted of a five-stage encoder and a four-stage decoder. Because the shallower decoder reduced reconstruction capacity, a wider latent representation was used than in the symmetric CAE. Two feature-expansion strategies were evaluated, differing in how rapidly the number of feature maps increased across encoder stages. The final configuration used Batch Normalization and LeakyReLU activations throughout the encoder and a 128-dimensional latent representation ($d_{z}=128$). This design implemented the intended combination of a deeper encoder, wider latent representation, and shallower decoder. An example reconstruction is shown in Figure 9.

A stacked autoencoder configuration was further evaluated to investigate whether noise suppression and anomaly-sensitive reconstruction could be separated into consecutive stages. The architecture contained two convolutional autoencoders,

$${\hat{X}}_{1}=AE_{1}\left(X_{p}\right)$$

followed by

$${\hat{X}}_{2}=AE_{2}\left({\hat{X}}_{1}\right).$$

Both components are based on four-stage grayscale convolutional autoencoders, but they use different bottleneck capacities. The first model is intentionally less constrained and uses a latent dimensionality of $d_{z,1}=256$, whereas the second model employs a narrower latent representation of $d_{z,2}=64$. The intended role of AE1 is to suppress difficult-to-reconstruct measurement variability while retaining the dominant spectrum structure, after which AE2 performs a more restrictive reconstruction.

In practice, however, the first autoencoder may suppress not only measurement noise but also anomalous structures that should remain detectable by the second reconstruction stage. To reduce this effect, an augmented stacked variant was introduced. For the augmented stacked configuration, the latent dimensions were $d_{z,1}=128$ and $d_{z,2}=32$.

After the first reconstruction ${\hat{X}}_{1}=AE_{1}\left(X_{p}\right)$, the positive pixel-wise deviation of the input from the reconstruction was computed as

$$Y=\max\left(X_{p}-{\hat{X}}_{1},\;0\right).$$

The intermediate representation supplied to the second autoencoder was then obtained as

$$X_{\mathrm{aug}}=\max\left(X_{p}-Y,\;0\right).$$

This transformation suppressed components of the original input that exceeded the first-stage reconstruction while retaining the remaining spectral structure. The second reconstruction was subsequently computed as

$${\hat{X}}_{2}=AE_{2}\left(X_{\mathrm{aug}}\right).$$

**Figure 10:**
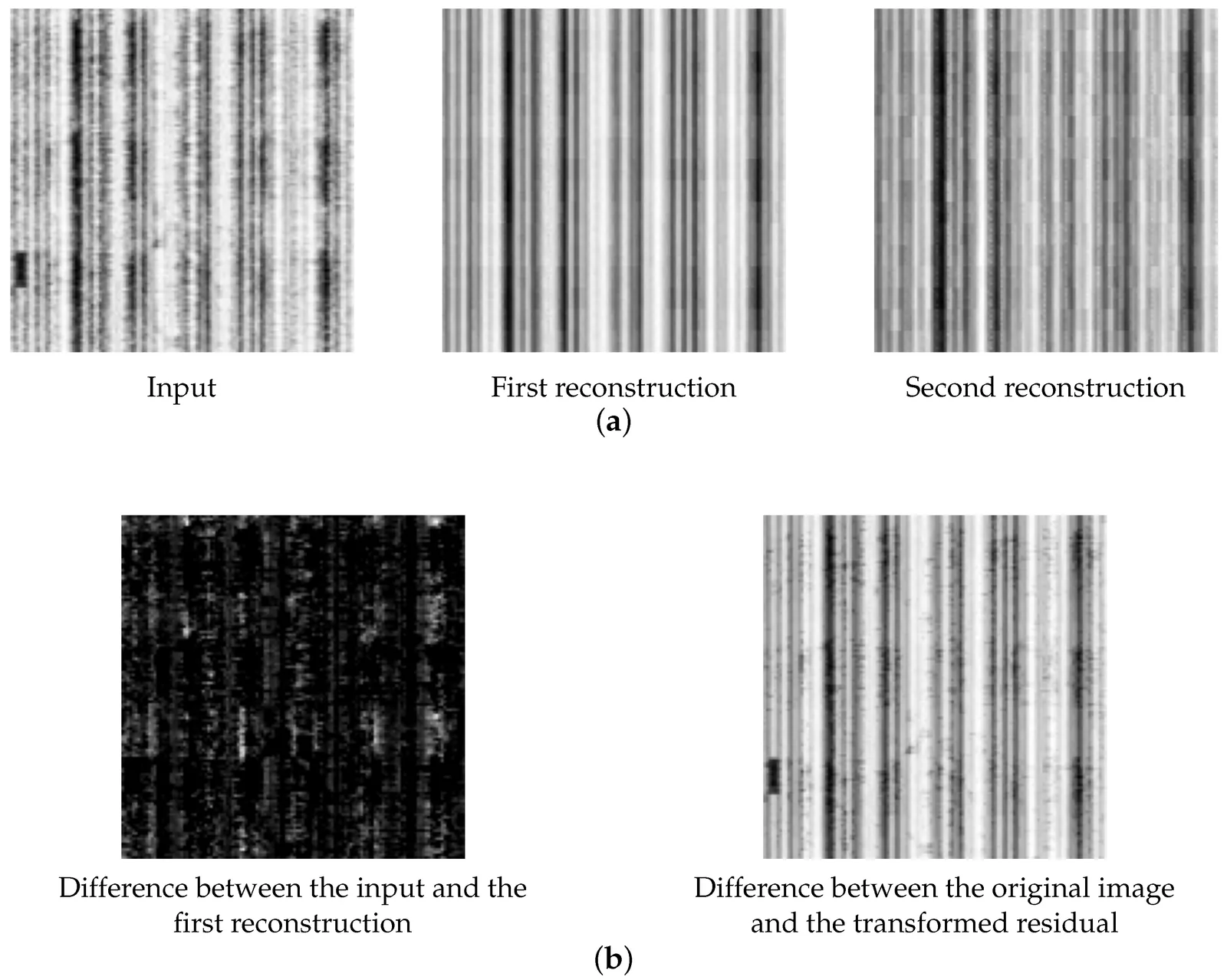
Stacked reconstruction strategies: (**a**) Direct stacked convolutional autoencoder, showing the input, the reconstruction of the first autoencoder, and the subsequent reconstruction of the second autoencoder. (**b**) Intermediate transformation used by the augmented stacked architecture, where the discrepancy obtained from the first reconstruction is used to suppress high-intensity disturbances before the second reconstruction stage.

The augmented representation was designed to reduce high-intensity reconstruction deviations before the second autoencoder while preserving structures that may remain relevant for anomaly detection. The direct and augmented stacked reconstruction procedures are illustrated in Figure 10.

For the axis-wise pixel representations introduced in Section 4.2, independent convolutional autoencoders were trained for the two sorting directions. For the sorted spectrum representations, smaller bottleneck dimensions were evaluated than for the principal unsorted reference configuration. The frequency-axis-sorted representation was processed using a three-stage convolutional autoencoder with a latent dimensionality of $d_{z,f}=8$, whereas the time-axis-sorted representation was processed using a four-stage convolutional autoencoder with $d_{z,t}=16$. For each observation, an anomaly score was calculated independently from the two reconstructions, and the final score was obtained by summing the two scores,

$$s_{\mathrm{sort}}\left(X\right)=s_{f}\left(X\right)+s_{t}\left(X\right).$$

Finally, a convolutional variational autoencoder (CVAE) was evaluated by replacing the deterministic bottleneck of the preprocessed CAE with a probabilistic latent representation. The convolutional encoder backbone remained conceptually consistent with the deterministic CAE, but its final representation was mapped to two vectors describing the parameters of a Gaussian latent distribution:

$$\mu =f_{\mu }\left(h\right),\;\ell =f_{\log\sigma^{2}}\left(h\right),\;\sigma =\exp\left(\frac{1}{2}\ell \right),$$

where *h* denoted the flattened convolutional representation. A latent sample was obtained through the reparameterization operation

$$z=\mu +\exp\left(\frac{1}{2}\ell \right)⊙\epsilon ,\;\epsilon ∼N\left(0,I\right).$$

The sampled vector *z* was subsequently passed to the convolutional decoder. This formulation preserved differentiability while constraining the latent representation toward a continuous probability distribution. The CVAE training objective combined the reconstruction loss with a weighted Kullback–Leibler regularization term,

$$L_{\mathrm{VAE}}=L_{\mathrm{rec}}+\beta_{\mathrm{KL}}L_{\mathrm{KL}}\;,$$

where

$$L_{\mathrm{KL}}=-\frac{1}{2}\sum_{k}\left(1+\log\sigma_{k}^{2}-\mu_{k}^{2}-\sigma_{k}^{2}\right).$$

During CVAE development, KL-divergence annealing was investigated using multiple weighting schedules in order to control the balance between reconstruction accuracy and latent-space regularization. Consequently, no single annealing schedule was treated as a fixed hyperparameter representative of all CVAE experiments.

The investigated KL-annealing schedules constituted an exploratory rather than exhaustive search of possible regularization settings. Accordingly, the observed reduction of the KL contribution should be interpreted as posterior collapse under the investigated CVAE configurations rather than as evidence that posterior collapse is intrinsic to CVAEs for FM-spectrum anomaly detection.

Across the evaluated CVAE configurations, the KL contribution tended toward very small values, consistent with posterior collapse under the investigated settings. Because the CVAE did not provide competitive quantitative performance, it is retained only as an exploratory negative result and is excluded from the principal configuration comparison.

Taken together, the evaluated architecture family modifies three principal sources of reconstruction capacity: network depth, latent-space dimensionality, and encoder–decoder balance. The symmetric CAE constrains the representation mainly through its bottleneck, the asymmetric model additionally limits reconstruction through decoder depth, the stacked models distribute the reconstruction task across consecutive stages, and the CVAE replaces the deterministic bottleneck with a probabilistic latent representation. This design enables a comparative exploration of how representational capacity, reconstruction capacity, and the form of the latent constraint are associated with anomaly-detection performance.

### 4.4. Reconstruction Residual and Anomaly Scoring

The detection of anomalies was based on the discrepancy between the preprocessed input $X_{p}$ and its reconstruction $\hat{X}$. The absolute residual map in pixels was defined as

$$R\left(i,j\right)=|X_{p}\left(i,j\right)-\hat{X}\left(i,j\right)|.$$

Several functions were evaluated to convert the reconstruction discrepancy between $X_{p}$ and $\hat{X}$ into a scalar anomaly score s(X), including MSE, SSIM, maximum residual, percentile, standard deviation, gradient-based discrepancy, and TopK aggregation. Residual-based measures operated on the absolute reconstruction-error map R, whereas SSIM directly evaluated structural similarity between the input and its reconstruction.

For percentile-based scoring, the absolute residual values were flattened into a single vector for each observation, and the anomaly score was defined as the 99th percentile of this distribution,

$$s_{perc}\left(X\right)=Q_{0.99}\left(R\right)$$

where $Q_{0.99}$ denoted the 99th percentile of the absolute reconstruction residuals. This formulation emphasizes large reconstruction discrepancies while being less dependent on a single extreme pixel than maximum-residual scoring.

For dispersion-based scoring, the anomaly score was calculated as the standard deviation of the flattened absolute residual values,

$$s_{std}\left(X\right)=std\left(\mathrm{vec}\left(R\right)\right)$$

where vec(R) denoted the vectorized absolute residual map. This score characterized the dispersion of reconstruction errors within an observation and assigned larger values when the residual magnitudes were more heterogeneous across the image.

For gradient-based scoring, Sobel gradient-magnitude maps were computed independently for the preprocessed input and its reconstruction using the horizontal and vertical kernels defined in Section 4.2.2. The corresponding gradient magnitudes were defined as

$$G_{X}\left(i,j\right)=\sqrt{G_{x,X}{\left(i,j\right)}^{2}+G_{y,X}{\left(i,j\right)}^{2}}$$

and

$$G_{\hat{X}}\left(i,j\right)=\sqrt{G_{x,\hat{X}}{\left(i,j\right)}^{2}+G_{y,\hat{X}}{\left(i,j\right)}^{2}}.$$

The gradient-based anomaly score was then calculated as the mean squared difference between the two gradient-magnitude maps,

$$s_{grad}\left(X\right)=\frac{1}{HW}\sum_{i=1}^{H}\sum_{j=1}^{W}{\left(G_{X}\left(i,j\right)-G_{\hat{X}}\left(i,j\right)\right)}^{2}.$$

This measure emphasized discrepancies in local structural transitions between the input and its reconstruction rather than differences in pixel intensity alone.

The scoring function associated with the best-performing configuration used the mean of the K largest residual values,

$$s_{TopK}\left(X\right)=\frac{1}{K}\sum_{r\in TopK(R)}r.$$

This formulation was less sensitive to isolated noisy pixels than the absolute maximum, while preserving localized high-magnitude residuals that would be suppressed by global averaging. The development-selected symmetric reference configuration used K=17, while *K* was treated as a configuration-specific development hyperparameter for the alternative models; the corresponding values are summarized in Table 2.

### 4.5. Training Procedure and Hyperparameter Selection

All deterministic autoencoders were optimized exclusively using normal spectrum observations, with reconstruction MSE as the training objective. The CVAE was trained using the reconstruction objective together with the weighted KL-divergence regularization defined in Section 4.3. Consequently, anomalous observations were not used for gradient-based parameter optimization in any of the investigated models. The Adam optimizer and a batch size of 8 were used throughout the experiments. The maximum training duration ranged from 30 to 50 epochs across the investigated configurations, as summarized in Table 3. Where applicable, training was controlled using early stopping together with a ReduceLROnPlateau learning-rate scheduler.

A separate labeled development set containing both normal and anomalous observations was used to monitor anomaly-detection performance during model development. The $F_{1}$-score on the labeled development set was used as the performance criterion for learning-rate scheduling, early stopping where applicable, checkpoint selection, architectural comparison, and operating-point calibration. Importantly, samples from the labeled development set were not used for gradient computation or reconstruction-loss optimization.

Architectural and preprocessing configurations were experimentally investigated, with particular emphasis on input resolution, network depth, latent dimensionality, encoder–decoder configuration, input representation and reconstruction-scoring strategy. The objective of this procedure was to compare the relative behavior of alternative reconstruction-based pipelines under a common development protocol rather than to estimate performance on an independently held-out deployment set.

The principal configuration-specific training hyperparameters are summarized in Table 3, while the anomaly-scoring and operating-point selection settings are reported separately in Table 2. A batch size of 8 was used for all configurations. Adam was used as the optimizer, while ReduceLROnPlateau decreased the learning rate by a factor of 0.1 when the monitored development-set $F_{1}$-score did not improve for the configuration-specific patience period. The values of the latent dimensionality, maximum number of epochs, early-stopping patience, and configuration-specific scoring parameters were selected experimentally during model development.

**Table 2:** Configuration-specific anomaly-scoring settings used for the principal model comparison. The listed scoring hyperparameters were selected using the $F_{1}$-score as the primary model-development criterion. For the final evaluation of each fixed configuration, decision thresholds were optimized separately for the $F_{1}$- and $F_{2}$-scores on the labeled development set.

Configuration	TopK *K*	Selection Metric
RGB baseline	–	$F_{1}$
Symmetric CAE	17	$F_{1}$
Asymmetric CAE	20	$F_{1}$
Stacked CAE	10	$F_{1}$
Augmented stacked CAE	20	$F_{1}$
Pixel-sorted CAE	17	$F_{1}$
CVAE	17	$F_{1}$

**Table 3:** Principal training hyperparameters used for the evaluated autoencoder configurations. ES denotes early-stopping patience and LR patience denotes the patience parameter of the ReduceLROnPlateau scheduler.

Configuration	Input	LatentDim.	Initial LR	Max.Epochs	ESPatience	LRPatience
RGB baseline	3×128×128	8	$10^{-2}$	30	7	2
Symmetric CAE	1×128×128	32	$10^{-2}$	30	7	2
Asymmetric CAE	1×128×128	128	$10^{-2}$	40	7	2
Stacked CAE	1×128×128	256/64	$10^{-3}$	50	10	3
Augmented stacked CAE	1×128×128	128/32	$10^{-3}$	30	9	4
Pixel-sorted CAE	1×128×128	8/16	$10^{-2}$	30	–	6
CVAE	1×128×128	32	$10^{-2}$	50	10	2

For the CVAE, the contribution of the KL-divergence term was controlled through KL-weight annealing. Multiple annealing schedules were investigated during model development, as described in Section 4.3; therefore, the CVAE experiments were not characterized by a single fixed annealing schedule.

All model-development decisions were completed before held-out testing. Specifically, preprocessing, network architecture, latent-space dimensionality, checkpoint selection, anomaly-scoring formulation, TopK parameter selection, and metric-specific operating thresholds were determined exclusively from the training and development subsets. No component of the anomaly-detection pipeline was selected or modified on the basis of held-out test performance.

### 4.6. Anomaly Decision and Threshold Selection

The scalar anomaly score was converted into a binary decision using a threshold τ. For each investigated configuration, metric-specific operating thresholds were determined on the labeled development set. The threshold used to report the $F_{1}$-score was selected as

$$\tau_{F_{1}}^{*}=\arg\max_{\tau }F_{1}\left(\tau \right),$$

whereas the threshold used to report the complementary recall-oriented $F_{2}$-score was selected independently as

$$\tau_{F_{2}}^{*}=\arg\max_{\tau }F_{2}\left(\tau \right).$$

The underlying model, preprocessing configuration, anomaly-scoring method, and associated scoring hyperparameters were kept identical when evaluating $F_{1}$ and $F_{2}$; only the decision threshold was optimized separately for the two metrics. The $F_{1}$-score served as the primary criterion for model development, architectural comparison, checkpoint selection, and scoring-hyperparameter selection, whereas $F_{2}$-threshold optimization was performed only to report the recall-oriented performance of the already selected configurations.

Because the same labeled development subset was used for model selection, scoring-hyperparameter selection, and operating-point calibration, the development-set results should be interpreted as model-development performance rather than as unbiased estimates of generalization. Independent generalization performance was assessed using the held-out test subset, with all model, scoring, and operating-point parameters frozen before evaluation.

### 4.7. Experimental Protocol and Evaluation Metrics

The experimental protocol comprised three functionally distinct subsets: a normal-only training subset, a labeled development subset, and an independently held-out labeled test subset. Autoencoder parameter optimization was performed exclusively using the normal-only training subset. The labeled development subset was used for $F_{1}$-guided model development, including model and checkpoint selection, architectural comparison, anomaly-scoring hyperparameter selection, and operating-point calibration. After all corresponding choices had been frozen, the held-out test subset was used exclusively for final generalization assessment.

Performance was quantified using precision, recall, $F_{1}$-score, and $F_{2}$-score. Precision *P* and recall *R* are defined as

$$P=\frac{TP}{TP+FP},$$

$$R=\frac{TP}{TP+FN},$$

where TP, FP, and FN denoted the numbers of true-positive, false-positive, and false-negative classifications, respectively. The $F_{1}$-score was the harmonic mean of precision and recall,

$$F_{1}=2\frac{PR}{P+R},$$

whereas the more general $F_{\beta }$-score was defined as

$$F_{\beta }=\left(1+\beta^{2}\right)\frac{PR}{\beta^{2}P+R}.$$

For β=2, the resulting $F_{2}$-score was

$$F_{2}=5\frac{PR}{4P+R}.$$

Both metric-specific operating thresholds were determined exclusively from the labeled development subset. Once the final model and scoring configuration had been selected, the resulting thresholds $\tau_{F_{1}}^{*}$ and $\tau_{F_{2}}^{*}$ were frozen. These same threshold values were subsequently applied unchanged to the independently held-out test subset. No threshold optimization, recalibration, or post hoc adjustment was performed using held-out test labels.

For held-out evaluation, precision, recall, $F_{1}$-score, accuracy, and the complete confusion matrix were calculated at the development-selected $F_{1}$ operating threshold. Threshold-independent AUROC and average precision (AP) were additionally calculated from the continuous anomaly scores for the held-out evaluation and for the controlled reconstruction-scoring comparison. These metrics were calculated without selecting or recalibrating a decision threshold on the held-out observations. No decision threshold was optimized, recalibrated, or post hoc adjusted using held-out test labels.

The $F_{2}$-score was retained as a complementary recall-oriented metric for the development-set comparison, using its separately optimized development-set threshold as described above. It was not used for held-out model ranking. Because the held-out subset was comparatively small, 95% confidence intervals for the held-out $F_{1}$-score were estimated using stratified bootstrap resampling.

## 5. Results

### 5.1. Effect of Preprocessing and Model Capacity

**Figure 11:**
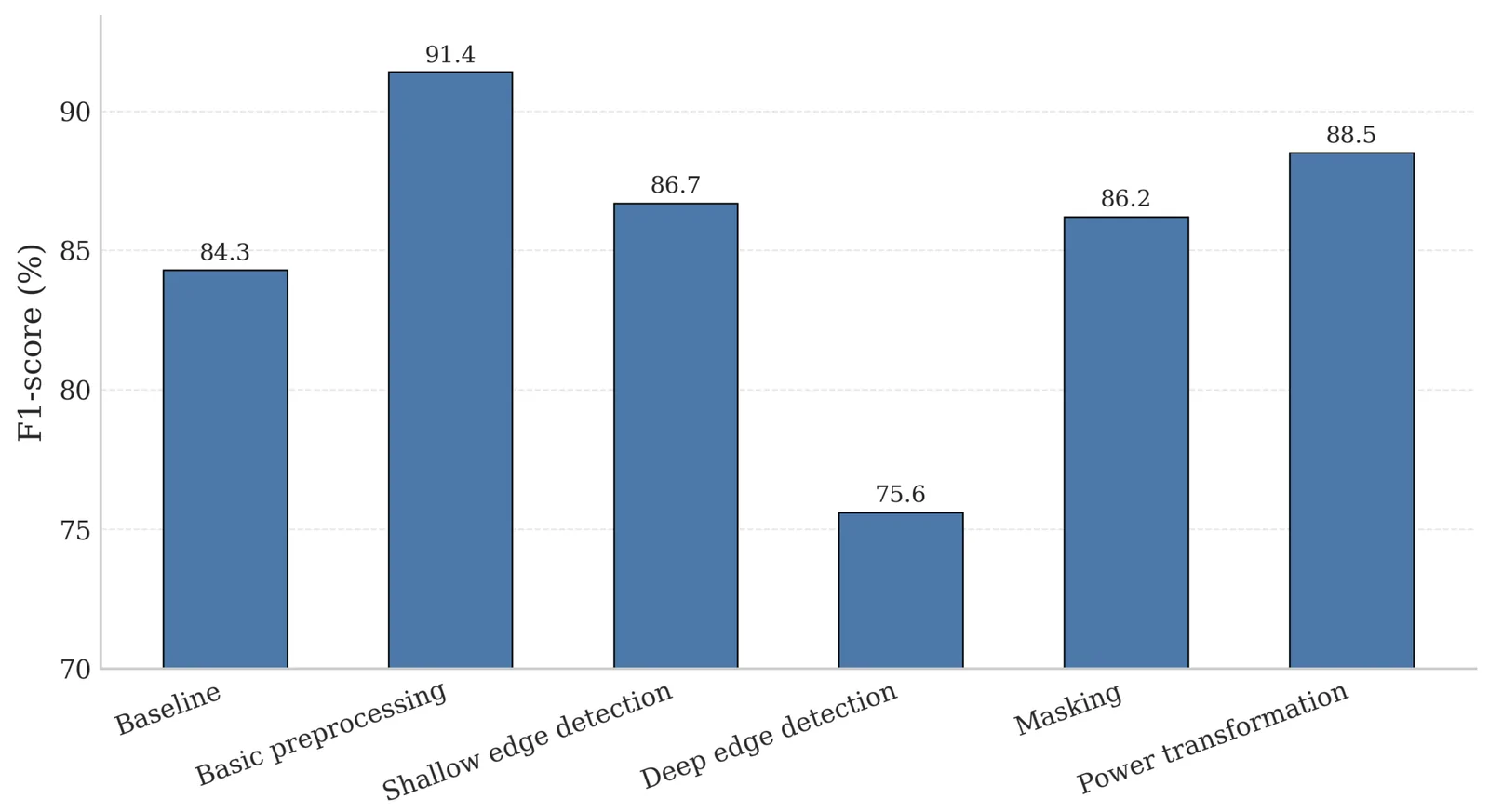
$F_{1}$-scores obtained by the evaluated preprocessing-related pipeline configurations. The grayscale, normalized, and downsampled symmetric CAE configuration achieved the highest observed score among the investigated variants.

The preprocessed reference pipeline achieved substantially higher detection performance than the original RGB baseline. The original RGB configuration achieved an $F_{1}$-score of 84.3%, whereas the grayscale, normalized, and downsampled symmetric CAE configuration reached 91.4%, corresponding to a 7.1 percentage-point difference between the two evaluated pipelines. Because the configurations also differed in bottleneck dimensionality and anomaly-scoring setup, this difference should be interpreted as a pipeline-level improvement rather than as the isolated effect of preprocessing alone. The additional intensity transformations did not further improve the preprocessed baseline representation: the power-transformed representation achieved an $F_{1}$-score of 88.5%, masking achieved 86.2%, and the shallow edge-augmented representation reached 86.7%. The deeper edge-based configuration performed considerably worse, reaching only 75.6%. The effect of the investigated preprocessing and spectrum-representation strategies on the $F_{1}$-score is summarized in Figure 11.

**Figure 12:**
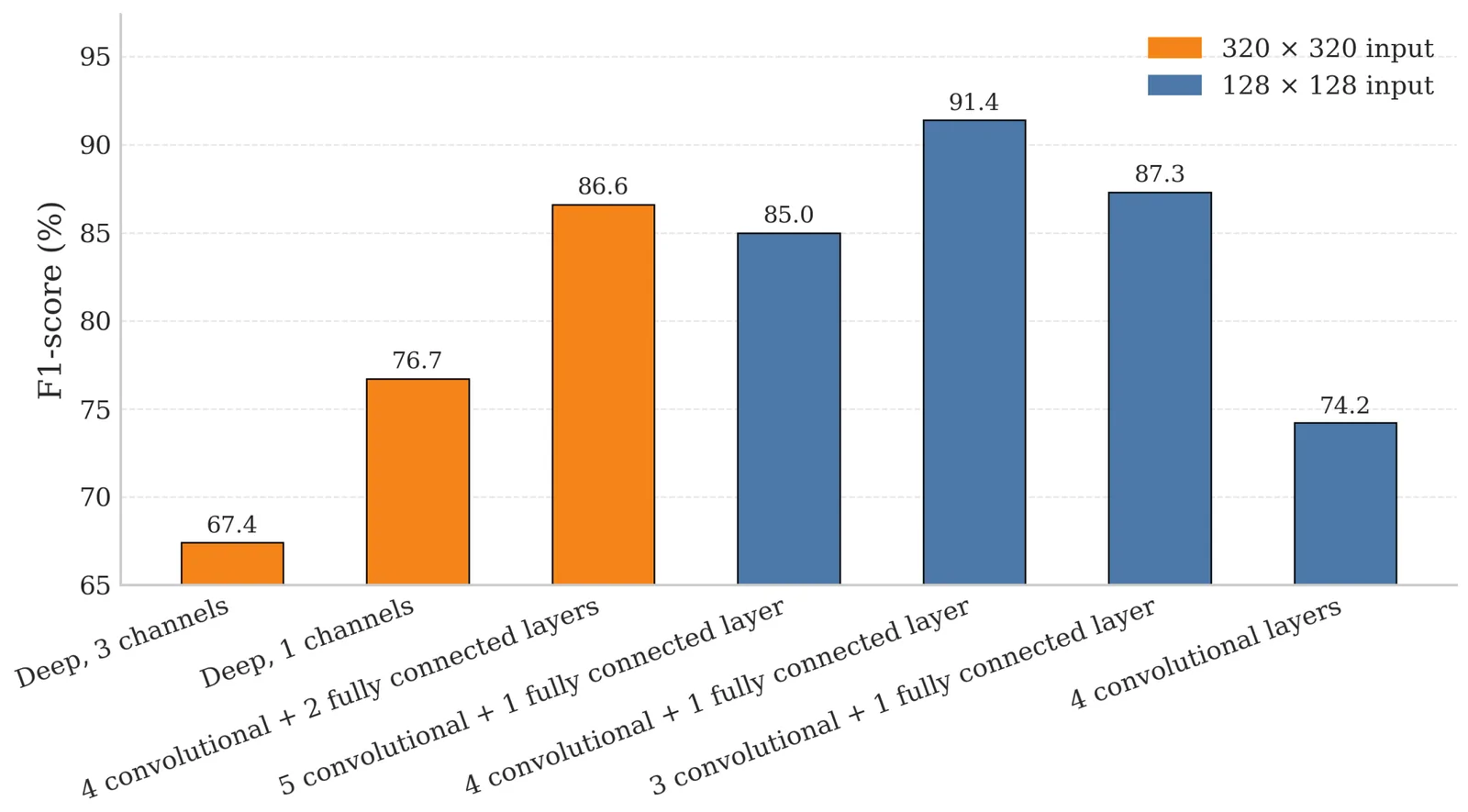
Effect of input resolution and autoencoder depth on $F_{1}$-score. The best performance was obtained using a 128×128 input and four convolutional layers followed by a single linear bottleneck.

Network capacity showed a similarly non-monotonic relationship with detection performance. Among the 128×128 models, the four-layer convolutional architecture with a single linear bottleneck achieved the highest $F_{1}$-score of 91.4%. Reducing the network to three convolutional layers decreased the score to 87.3%, whereas increasing it to five layers resulted in 85.0%. Removing the explicit linear bottleneck reduced the score to 74.2%. The larger 320 × 320 architectures also failed to outperform the smaller model, with scores ranging from 67.4% to 86.6%. The relationship between input resolution, network depth, and detection performance is summarized in Figure 12.

### 5.2. Controlled Reconstruction-Scoring Ablation

To isolate the contribution of the anomaly-scoring formulation from differences in reconstruction quality, a controlled scoring ablation was performed using a single frozen symmetric CAE. The preprocessing, network architecture, learned parameters, input observations, and reconstructed images were therefore identical for all scoring methods. Only the scalar function used to transform the reconstruction discrepancy into an observation-level anomaly score was varied.

The evaluated scoring methods were MSE, structural dissimilarity (1−SSIM), maximum absolute residual, the 99th percentile of the absolute residual distribution, residual standard deviation, gradient-based discrepancy, and TopK residual aggregation with K=17. Because this controlled analysis focused on score separability without introducing score-specific test-set threshold optimization, AUROC and average precision (AP) are reported as threshold-independent metrics. The corresponding threshold-independent performance results are summarized in Table 4.

Under the controlled setting, maximum-residual scoring achieved the highest threshold-independent discrimination, with an AUROC of 0.827 and an AP of 0.858. TopK aggregation with K=17 remained competitive, reaching an AUROC of 0.803 and an AP of 0.847, but it did not outperform maximum-residual scoring on the held-out observations. Consequently, the controlled experiment does not support a universal causal advantage of TopK scoring. The 91.4% development $F_{1}$-score obtained in the original model-development experiments should instead be interpreted as the performance of the complete selected pipeline, including its preprocessing, reconstruction model, scoring formulation, and operating-point selection.

### 5.3. Sensitivity to the TopK Aggregation Parameter

The TopK parameter was treated as a development hyperparameter in the original model-development experiments. To provide the additional sensitivity analysis requested for this parameter, a post hoc experiment was performed using a fixed archived symmetric CAE checkpoint and the original labeled development subset. No model parameters were modified during this analysis.

The purpose of this experiment was to characterize the dependence of detection performance on the aggregation size *K*, rather than to reproduce or replace the original model-selection results reported in Section 5.4. Because the sensitivity experiment was performed after the principal development experiments using the available archived checkpoint, its absolute $F_{1}$ values are therefore reported separately from the original configuration-level results.

For a 128×128 residual map, the originally selected value K=17 corresponds to approximately 0.104% of the 16,384 residual pixels. The archived symmetric CAE was evaluated over K∈{1, 2, 5, 10, 15, 17, 20, 25, 30, 50, 100, 200, 500}. For each value of *K*, the image-level anomaly score was recomputed and the corresponding $F_{1}$-optimized operating point was determined on the labeled development subset.

The post hoc sensitivity analysis shows that detection performance was comparatively insensitive to the exact aggregation size over a broad range of *K*. Within this fixed-checkpoint experiment, the $F_{1}$-score varied only from 85.0% to 85.7% for *K* values between 5 and 500, with the highest point estimate obtained at K=25. The originally selected K=17 produced an $F_{1}$-score of 85.1% in this post hoc experiment. The complete sensitivity results for the investigated TopK aggregation sizes are summarized in Table 5.

These values should not be interpreted as a re-estimation of the original principal development result of 91.4%. The sensitivity experiment was conducted separately using a fixed archived checkpoint and was introduced specifically to evaluate robustness to the TopK aggregation size. Its relevant finding is therefore the comparatively flat dependence on *K*, rather than the absolute score of any individual operating point.

Because pixel-level anomaly masks were unavailable, *K* should not be interpreted as the physical spatial extent of an anomaly. Rather, it controls the number of largest reconstruction residuals contributing to the observation-level anomaly score. The results indicate that the benefit of upper-tail residual aggregation is not dependent on a single narrowly defined value of *K*.

### 5.4. Configuration-Level Development Performance

**Figure 13:**
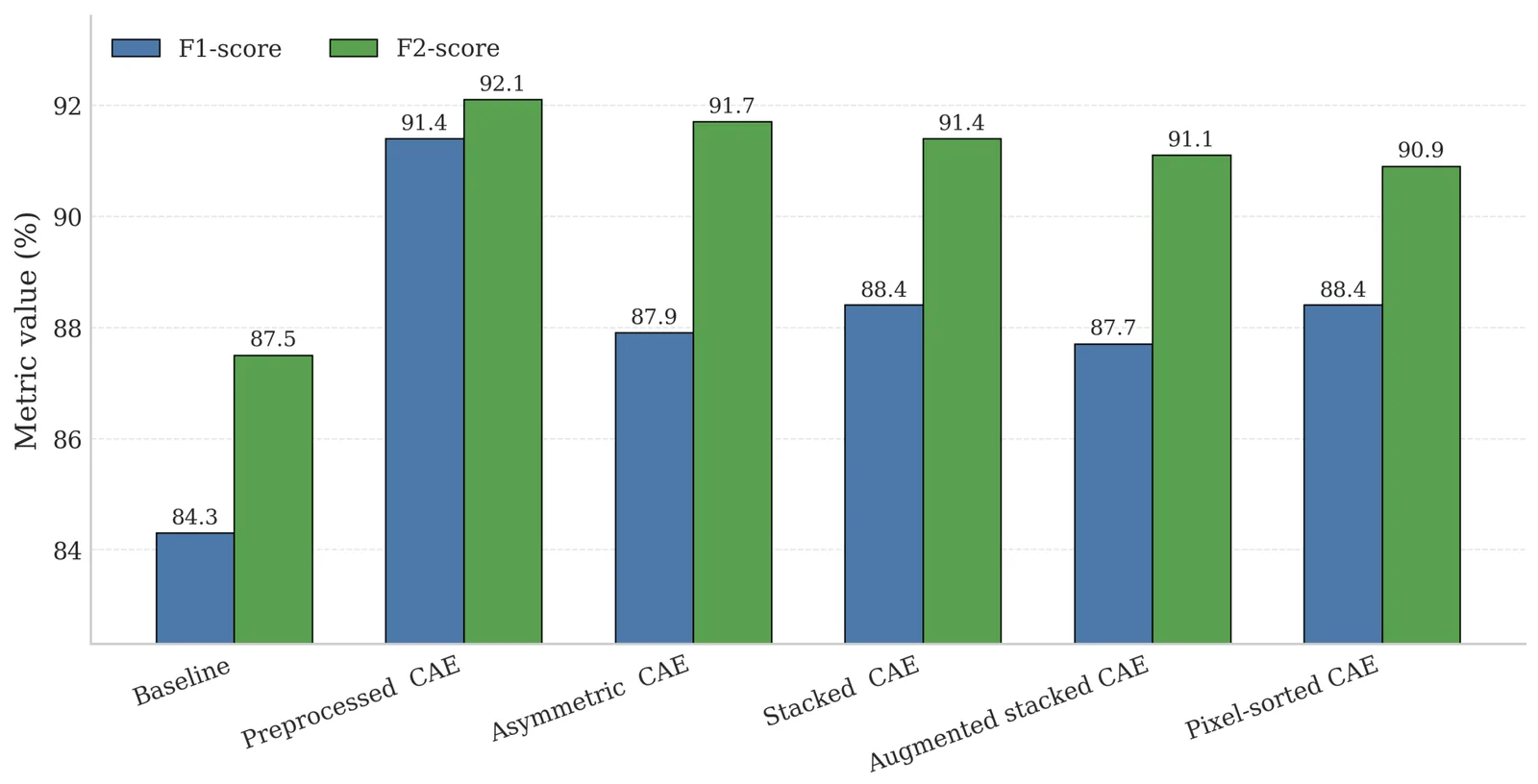
Comparison of the principal anomaly-detection configurations in terms of $F_{1}$- and $F_{2}$-scores. The same model and anomaly-scoring hyperparameters were used for both metrics within each configuration, while the decision threshold was optimized separately for $F_{1}$ and $F_{2}$ on the labeled development set. The reported values therefore represent metric-specific optimized operating points.

Table 2 and Figure 13 summarize the observed development-set performance of the principal reconstruction pipelines. For all configurations, model and scoring hyperparameters were selected using the $F_{1}$-score as the primary development criterion. For the final metric calculation, the decision threshold was optimized separately for $F_{1}$ and $F_{2}$ on the labeled development set. Consequently, the $F_{1}$-scores reported below correspond to $F_{1}$-optimized operating points, whereas the $F_{2}$-scores correspond to independently optimized $F_{2}$ operating points for the same underlying model and scoring configurations. The original RGB baseline achieved $F_{1}$ and $F_{2}$ scores of 84.3% and 87.5%, respectively. The symmetric CAE using the proposed baseline preprocessing achieved the highest $F_{1}$-score of 91.4% at its $F_{1}$-optimized operating point and an $F_{2}$-score of 92.1% at its separately $F_{2}$-optimized operating point.

The alternative architectures produced similar but consistently lower $F_{1}$-scores. The asymmetric CAE achieved 87.9%$F_{1}$ and 91.7%$F_{2}$, while the stacked and augmented stacked models achieved 88.4%/91.4% and 87.7%/91.1%, respectively. The pixel-sorted approach reached 88.4%$F_{1}$ and 90.9%$F_{2}$. Thus, although the more specialized architectures remained competitive in recall-oriented $F_{2}$ performance, none exceeded the comparatively simple symmetric preprocessed CAE.

The asymmetric and stacked architectures exhibited relatively higher $F_{2}$ values than $F_{1}$ values, indicating that their precision–recall balance resulted in comparatively stronger recall-weighted performance.

The convolutional VAE was omitted from the final comparison because its performance was substantially below that of the deterministic autoencoder variants. The experiments additionally showed a progressive reduction of the KL-divergence term toward zero, consistent with posterior collapse and a weak dependence of the latent representation on the individual input. The CVAE was therefore excluded from the quantitative comparison in Figure 13, while its observed training behavior is discussed qualitatively in Section 6.

These values represent configuration rankings obtained during model development. Because model and operating-point selection were performed using the same labeled development subset, small differences between configurations should not be interpreted as definitive evidence of generalization superiority.

### 5.5. Held-Out Test Performance

Following completion of model development, the frozen configurations were evaluated on the independently held-out subset containing 37 normal and 53 anomalous observations. No model architecture, checkpoint, scoring hyperparameter, TopK value, or $F_{1}$ decision threshold was modified on the basis of held-out performance.

Precision, recall, $F_{1}$-score, accuracy, and confusion-matrix counts were calculated using the development-selected $F_{1}$ operating point. Thus, the reported test results represent direct application of the development decision rule rather than test-set-optimized operating points.

Table 6 shows that the development-set ranking did not transfer unchanged to the held-out subset. The asymmetric CAE achieved the highest held-out $F_{1}$ point estimate, while the other principal configurations exhibited lower but overlapping performance ranges. The corresponding held-out confusion-matrix counts are reported in Table 7. These results reinforce the need to distinguish development-set model-selection performance from independent generalization performance and indicate that relatively small differences observed during development should not be interpreted as definitive evidence of architectural superiority.

The held-out evaluation provides two complementary views of generalization. Table 6 reports threshold-dependent performance at the development-selected operating points, whereas Table 8 reports threshold-independent discrimination using AUROC and average precision. The development-set ranking did not transfer unchanged to the held-out subset. The asymmetric CAE achieved the highest held-out $F_{1}$ point estimate, whereas the pixel-sorted CAE achieved the strongest threshold-independent discrimination, with an AUROC of 0.895 and an AP of 0.942. These differences further indicate that conclusions about configuration superiority depend partly on the selected operating criterion and reinforce the need to distinguish development-set model-selection performance from independent generalization performance.

A common characteristic of the held-out results was high anomaly recall combined with substantially lower precision. In particular, the asymmetric CAE detected 52 of the 53 anomalous observations, corresponding to 98.1% recall, but it produced 23 false-positive alarms among the 37 normal observations. Thus, the principal generalization limitation of the evaluated systems is not missed anomalies but the comparatively high false-positive rate under unseen conditions.

## 6. Discussion

Taken together, the experiments indicate that anomaly-detection performance is governed by the balance between information preservation and constrained reconstruction rather than by model complexity alone. The stronger performance of the preprocessed reference pipeline, together with the deterioration observed for deeper or higher-resolution variants, suggests that reducing nuisance variability while maintaining a moderately constrained reconstruction model can be beneficial. However, because preprocessing and bottleneck capacity were not varied independently in all the comparisons, the contribution of preprocessing should be interpreted jointly with the associated model configuration. This interpretation is consistent with the general limitation of reconstruction-based anomaly detection that overly expressive models may also reconstruct previously unseen abnormal structures, thus reducing the residual contrast between normal and anomalous observations [12].

The controlled reconstruction-scoring ablation confirms that observation-level scoring formulation materially affects anomaly separability even when the underlying reconstructions are identical. However, the controlled results provide a more nuanced interpretation than the original pipeline-level comparison. Maximum-residual scoring achieved the highest threshold-independent held-out performance (AUROC = 0.827, AP = 0.858), whereas TopK aggregation with K=17 achieved AUROC = 0.803 and AP = 0.847. Thus, the results do not establish a universal advantage of TopK over alternative scoring formulations.

Nevertheless, the post hoc TopK sensitivity analysis showed that upper-tail residual aggregation remained comparatively stable over a broad range of *K* values. Taken together, these observations suggest that localized reconstruction discrepancies are informative for the investigated FM-spectrum anomalies, while the precise aggregation rule and operating point depend on the reconstruction model and experimental configuration.

Several limitations should be considered when interpreting the revised results. First, although the introduction of a separately held-out test subset removed the direct re-use of development observations for final generalization assessment, the held-out subset contained only 90 observations. Consequently, small numerical differences between configurations should be interpreted cautiously and together with their sampling uncertainty rather than as definitive model rankings.

Second, the held-out results indicate a substantial reduction in precision relative to recall. The evaluated detectors retained very high sensitivity to anomalous observations, but several normal observations were classified as anomalous. In an operational monitoring environment, this behavior could result in an excessive false-alarm rate and therefore represents an important limitation for direct autonomous deployment.

Third, the investigated measurements originate from a specific FM-spectrum monitoring environment. Further external validation across additional monitoring locations, acquisition periods, measurement conditions, instruments, and anomaly types is therefore necessary to establish broader deployment-level robustness. Importantly, such future calibration should be performed using new development data rather than by adjusting the operating threshold on the held-out subset used in the present study.

## 7. Conclusions

This study demonstrates that reconstruction-based FM-spectrum anomaly detection is influenced by the complete processing pipeline rather than by autoencoder complexity alone. During model development, the strongest configuration combined grayscale normalization and downsampling with a symmetric convolutional autoencoder and localized residual scoring, achieving an $F_{1}$-score of 91.4% on the labeled development subset.

The introduction of a separately held-out test subset provides a more realistic assessment of generalization. In the current held-out evaluation, the architecture ranking differed from that observed during development: the asymmetric CAE achieved the highest test $F_{1}$-score, whereas the symmetric CAE remained the strongest development configuration. This result demonstrates that relatively small differences observed during development should not be interpreted as definitive evidence of architectural superiority. The asymmetric CAE achieved the highest held-out $F_{1}$-score of 81.3%, compared with 79.1% for the symmetric CAE.

The controlled reconstruction-scoring ablation further showed that score formulation materially affects anomaly separability when reconstruction quality is held fixed. Maximum-residual scoring achieved the strongest threshold-independent discrimination in the controlled experiment, while TopK aggregation remained competitive. A separate post hoc sensitivity analysis further showed that performance was comparatively stable across a broad range of TopK aggregation sizes, indicating that the observed behavior was not dependent on a single narrowly tuned value of *K*.

Across the held-out configurations, anomaly recall remained high, while precision was reduced by a comparatively large number of false-positive detections. Consequently, the present approach appears more suitable as a monitoring-support or screening mechanism than as a fully autonomous alarm system in its current form. Further work should focus on improving normal-condition generalization and false-alarm control, followed by external validation across additional monitoring sites, acquisition periods, and anomaly types.

## Figures and Tables

**Table 1:** Composition and functional role of the training, development, and independently held-out test subsets. The development subset was used for model and operating-point selection, whereas the held-out test subset was used exclusively for final generalization assessment.

Subset	Normal	Anomalous	Total
Training	1787	0	1787
Development	138	141	279
Held-out test	37	53	90

**Table 4:** Controlled reconstruction-scoring comparison using identical reconstructions from a single frozen symmetric CAE.

Scoring Method	AUROC	AP
MSE	0.755	0.823
1−SSIM	0.802	0.847
Maximum residual	0.827	0.858
99th percentile	0.801	0.852
Residual standard deviation	0.762	0.797
Gradient discrepancy	0.647	0.775
TopK (K=17)	0.803	0.847

**Table 5:** Post hoc development-set sensitivity of the $F_{1}$-score to the TopK aggregation parameter using a fixed archived symmetric CAE checkpoint. For each value of *K*, only the TopK aggregation size and the corresponding development-set decision threshold were varied; the network parameters remained fixed.

*K*	Residual Pixels (%)	$F_{1}$ (%)	Threshold
1	0.006	84.1	0.51638
2	0.012	84.1	0.50904
5	0.031	85.2	0.47925
10	0.061	85.2	0.46677
15	0.092	85.4	0.46330
17	0.104	85.1	0.45818
20	0.122	85.2	0.44881
25	0.153	85.7	0.46892
30	0.183	85.5	0.43620
50	0.305	85.5	0.41481
100	0.610	85.0	0.37501
200	1.221	85.2	0.36071
500	3.052	85.1	0.29393

**Table 6:** Held-out test performance at the development-selected operating point. The 95% confidence intervals refer to the $F_{1}$-score and were estimated using stratified bootstrap resampling.

Configuration	Precision	Recall	$F_{1}$	Accuracy	$F_{1}$ 95% CI
RGB baseline	59.8%	98.1%	74.3%	60.0%	71.9–76.3%
Symmetric CAE	65.4%	100.0%	79.1%	68.9%	76.3–82.2%
Asymmetric CAE	69.3%	98.1%	81.3%	73.3%	77.3–85.5%
Augmented stacked CAE	63.1%	100.0%	77.4%	65.6%	75.2–80.3%
Pixel-sorted CAE	62.4%	100.0%	76.8%	64.4%	74.6–79.1%

**Table 7:** Held-out confusion-matrix counts at the development-selected $F_{1}$ operating point.

Configuration	TN	FP	FN	TP
RGB baseline	2	35	1	52
Symmetric CAE	9	28	0	53
Asymmetric CAE	14	23	1	52
Augmented stacked CAE	6	31	0	53
Pixel-sorted CAE	5	32	0	53

**Table 8:** Threshold-independent performance of the principal configurations on the independently held-out test subset. AUROC and average precision (AP) were calculated directly from the continuous anomaly scores without re-optimizing a decision threshold on the held-out observations.

Configuration	AUROC	AP
RGB baseline	0.484	0.631
Symmetric CAE	0.803	0.847
Asymmetric CAE	0.780	0.808
Augmented stacked CAE	0.811	0.863
Pixel-sorted CAE	0.895	0.942

## Data Availability

The FM spectrum measurement dataset analyzed in this study is publicly available on Zenodo, as described in [8]. The source code used for data preprocessing, autoencoder training, reconstruction-based anomaly scoring, and evaluation is also publicly available on Zenodo at https://doi.org/10.5281/zenodo.21902493 [21].
